# Effect of antiretrovirals on renal function in people taking HIV pre-exposure prophylaxis*

**DOI:** 10.1590/1518-8345.7280.4734

**Published:** 2025-11-17

**Authors:** Priscila Silva Pontes-Pereira, Rodrigo de Carvalho Santana, Elucir Gir, Renata Karina Reis

**Affiliations:** 1 Universidade de São Paulo, Escola de Enfermagem de Ribeirão Preto, PAHO/WHO Collaborating Centre for Nursing Research Development, Ribeirão Preto, SP, Brazil.; 2 Universidade de São Paulo, Faculdade de Medicina de Ribeirão Preto, Ribeirão Preto, SP, Brazil.

**Keywords:** Pre-Exposure Prophylaxis, Anti-Retroviral Agents, Tenofovir, Kidney Diseases, Physiologic Monitoring, Glomerular Filtration Rate

## Abstract

to evaluate the effect of antiretrovirals on renal function in individuals receiving HIV pre-exposure prophylaxis.

a descriptive, longitudinal survey design was used in a retrospective cohort study for 48 weeks. The sample consisted of 203 participants. Non-probabilistic convenience sampling was used. Sociodemographic, clinical, and laboratory variables were analyzed. Absolute and relative frequencies, mean, standard deviation, minimum and maximum values, Fisher’s exact test, and Friedman test were used, with a significance level of <5%.

the participants were predominantly cisgender men (86.2%), white (69.1%), highly educated (76.7%), and had a mean age of 34 years. The presence of markers of renal damage was low. Proteinuria increased until the 12th week (2.6%), maintaining a small variation until the 48th week. The decline in estimated Glomerular Filtration Rate (eGFR) ≥25% from baseline was 4.4%, 3%, 4.4%, and 3% at weeks 12, 24, 36, and 48, respectively. There was no significant association between eGFR decline ≥25% and the presence of any of the markers of renal damage.

the low incidence of events characterizing renal dysfunction, such as eGFR decline ≥25% and the presence of mild and isolated events of proteinuria, glycosuria, uricosuria, and cylindruria, was confirmed at 48 weeks of follow-up.

## Highlights

**(1)** Few studies on renal dysfunction in HIV pre-exposure prophylaxis users. **(2)** Low incidence of renal dysfunction in PrEP users during 48 weeks. **(3)** Low incidence of markers of renal damage during 48 weeks of follow-up. **(4)** Nurses’ clinical knowledge for monitoring renal function is essential.

## Introduction

The weakening progress in the response to the Human Immunodeficiency Virus (HIV) in recent years reflects a worrying reemergence of the HIV epidemic^(1)^. Global reports point to a total of 1.3 million new HIV infections in 2023^(2)^. If these trends continue, 1.2 million people will be infected by the virus in 2025, three times the target for that year^(3)^.

According to the targets proposed by the United Nations for achieving the Sustainable Development Goals (SDGs), the goal is to reduce the incidence of new HIV cases worldwide to 200,000 and eliminate the AIDS epidemic as a public health problem by 2030^(2)^. In this context, a shift in the paradigm of preventing sexual transmission of HIV has been observed due to advances made in recent years with the proposal for combined prevention. This strategy involves the recommendation and provision of several strategies, with emphasis on biomedical interventions based on the use of antiretroviral drugs, such as HIV Pre-Exposure Prophylaxis (PrEP), considered one of the most important recent advances in the prevention of HIV infection^(4)^. PrEP is based on a combination of antiretrovirals, one of the most common being the combination of tenofovir disoproxil fumarate (TDF) and oral emtricitabine (FTC) at a fixed dose of 300/200 mg^(4)^.

Despite the many benefits of regular PrEP use, users should be monitored for potential adverse effects^(5)^. It is worth noting that the literature has already established that TDF is associated with proximal renal tubular injury, crystal-induced obstruction or interstitial nephritis^(6)^, toxic acute tubular necrosis with dysmorphia, and distinct mitochondrial enlargement^(7)^. Clinical trials have shown that it is associated with an increased risk of acute kidney injury (AKI), nephrogenic proteinuria, Diabetes Insipidus, Fanconi syndrome, and chronic kidney disease (CKD)^(8-9)^.

Therefore, the concern about renal dysfunction among people using PrEP is relevant to clinical practice. Various studies describe a decline in the estimated glomerular filtration rate (eGFR) by TDF in people using PrEP, ranging from 1% to 15%^(10-14)^. This wide variability may be linked to the different population contexts investigated, including gender, ethnicity, and age^(10-14)^. Other indicators of renal injury have been reported, such as a decline in eGFR ≥25% compared to baseline^(15)^; and the presence of markers of proximal tubulopathy such as creatinine, non-albumin-containing proteinuria, euglycemic glycosuria, increased urinary phosphate excretion, and increased urinary uric acid excretion^(15-17)^.

Careful assessment of renal adverse effects is essential to maximize benefits and minimize risks^(9)^. Routine monitoring of renal function is critical in individuals receiving TDF, as continued use of a nephrotoxic antiretroviral drug will be included in these individuals’ daily routine as a prophylactic method for HIV prevention^(9)^. Given the presented topic and the proven international relevance of the agenda related to the action of a nephrotoxic antiretroviral used as a prophylactic agent for HIV prevention, from the indication of the preventive method to the frequency of assessment, and the impacts on medication adherence, clinical and economic aspects, justify the current interest among scientists and clinical nurses in reaching consensus on the use of PrEP in the renal system. Therefore, the objective of the presented study was to evaluate the effect of antiretrovirals on the renal function of people using PrEP.

## Method

### Study design

This is a retrospective cohort study conducted on HIV-negative individuals using PrEP for 48 weeks. The 48-week follow-up period for individuals using PrEP corresponded to those who initiated its use between January 2018 and January 2021. Data collection took place over a four-month period, from May to August 2022.

### Data collection setting

Eligible participants were identified among users of four Specialized Care Services (SCS) corresponding to the central, eastern, southern and northern regions located in southeastern Brazil, specifically in a municipality in the interior of the state of São Paulo.

### Population

Initially, 614 people were identified in the SCS who started using PrEP between January 2018 and January 2021, distributed across the SCS in the following regions: central (419), east (52), south (61) and north (64).

### Selection criteria

The selection criteria for this study included: individuals using PrEP and aged ≥18 years; availability of medical records for at least one follow-up visit and four quarterly follow-up visits, as well as laboratory test results; initiation of PrEP between January 2018 and January 2021; normal renal function (serum creatinine ≤1.3 mg/dL for men / ≤1.1 mg/dL for women and eGFR ≥60 mL/min) before starting PrEP. The exclusion criteria were: failure to complete the 48-week regular follow-up period; lack of complete records of information and laboratory results in the participants’ medical records; and a diagnosis of diabetes mellitus (DM).

### Sample definition

The sample was non-probabilistic, consecutive, and comprised all PrEP users who initiated its use between January 2018 and January 2021. The sample size included the eligible population during the data collection period; that is, all records in the medical records of people using PrEP were consulted and assessed for the study’s eligibility criteria manually, in a non-probabilistic manner, resulting in a total of 203 participants, belonging to the following SCSs regions: central (158), east (20), south (14), and north (11). It is worth noting the occurrence of missing data in the participants’ medical records throughout the 48 weeks of follow-up.

### Study variables

The variables considered for this study were sociodemographic, general clinical, and laboratory variables.

The sociodemographic variables included: sex at birth (female, male), sexual orientation (heterosexual, homosexual, bisexual, unknown); gender identity (cisgender man, transgender man, cisgender woman, transgender woman, transvestite, pansexual, other); age in years; age range (20-39 years/40-49 years/50 years); self-reported skin color (white, black, Asian, mixed race, not stated); education (none/no formal education/1-3 years of schooling/4-7 years of schooling/8-11 years of schooling/12 years of schooling); homelessness (yes, no).

Regarding general clinical and laboratory variables, the following were considered for the study: history of systemic arterial hypertension (SAH) (yes, no); dyslipidemia (yes, no); use of antihypertensive medications (yes, no); serum creatinine level (mg/dL); eGFR; urinalysis for proteinuria [absent, present-trace, one cross (1+), two crosses (2+), three or more crosses (3+)], glycosuria [(absent, present-(1+), two crosses (2+), three or more crosses (3+)], uric acid (present, absent), hyaline casts (present, absent), and granular casts (present, absent).

### Instruments used to collect information

For data collection, a semi-structured questionnaire was used. It underwent theoretical face and content validation by five experts in the field, who evaluated its relevance, ease of understanding, and relevance to the selected variables.

Both the data collection questionnaire and the validation questionnaire were emailed to five experts in infectious diseases, nephrology, and methodology, identified through their CVs, with a deadline of 15 days. After analysis and necessary adjustments, a pilot study was conducted with 15 medical records of people using PrEP in one of the SCSs. This instrument was used specifically to achieve the objectives of this study. It is worth noting that the 15 medical records from the pilot test were not included in the final study sample. To assess renal function, eGFR was calculated using empirical mathematical equations based on serum creatinine levels: Chronic Kidney Disease Epidemiology Collaboration (CKD-EPI) (mL/min/1.73 m^2^)^(18)^ and Cockcroft-Gault (CG) (mL/min)^(19)^, using the calculator in the National Kidney Foundation (NKF) app^(20)^.

To assess the severity scale of adverse events, specifically markers of kidney damage (proteinuria, glycosuria, and cylindruria), the severity scale for adverse events in adults used by *France Recherche Nord et Sud Sida-HIV et Hepatites* [National Agency for Research on AIDS and Hepatitis (ANRS)] was used, which grades events as: Grade 1 or mild event (trace to 1+); Grade 2 or moderate event (2+); and Grade 3 or severe event (3+ or more)(21). To identify renal function decline, we used the acronym RIFLE: risk (R), injury (I), failure (F), loss (L), and end-stage kidney disease (E). RIFLE establishes that the first three most sensitive classes, referring to the severity of renal dysfunction, are assessed by relative changes in serum creatinine levels or GFR from a baseline value^(22)^. As a marker of clinically significant renal injury, a significant decline in eGFR ≥25% compared to baseline levels at any time point was considered the cutoff point^(22)^.

### Data collection

Data were collected by the researcher and a research assistant after theoretical and practical training. Data collection was divided into five time points: baseline (before starting PrEP) and four quarterly clinical and laboratory follow-ups, totaling a 48-week follow-up period. Information on sociodemographic, general clinical, and laboratory test data was obtained, and eGFR was calculated using the CKD-EPI and CG equations.

Data were collected through the PrEP user registration form, the first appointment form, the 30-day follow-up form, the PrEP clinical follow-up form, and quarterly laboratory tests recorded in the Hygiaweb system, a health management software used in the municipality where the SCSs are located. It was developed to automate processes and promote integration between public health units. Its functions include providing online results of laboratory tests performed in health services.

To obtain eGFR using the CKD-EPI and CG equations, information on sex, age, and serum creatinine was required; and gender, age, weight, and serum creatinine, respectively. To identify markers of kidney damage, we evaluated the results of a single morning urine sample, which was analyzed quarterly, including: chemical parameters: uric acid; and urinary biomarkers: proteinuria and glycosuria (crosses) and cylindruria - hyaline casts and granular casts (present and absent). In our study, the proteinuria identified in laboratory tests was qualitative, classified as crosses (traces, 1+, 2+, 3+, and 4+).

### Data processing and analysis

Study data were collected and managed using Research Electronic Data Capture (REDCap) electronic data capture tools hosted and made available by the proposing institution^(23)^. REDCap is a secure, web-based software platform designed to support data capture for research studies, providing an intuitive interface for validated data capture and audit trails to track data manipulation and procedures^(23)^.

Data were processed and analyzed using the Statistical Package for the Social Sciences (SPSS) software, version 26.0. Descriptive statistics were used using absolute (n) and relative (%), mean, standard deviation (SD), minimum and maximum frequencies, and Fisher’s exact test of association. For significant variables, logistic regression was used to calculate the odds ratio (OR). Fisher’s exact test was also used to measure the association between reduced renal function and markers of renal damage. The Kolmogorov-Smirnov test for normality was applied to measure the symmetry between the CKD-EPI (mL/min/1.73 m^2^) and Cockcroft-Gault (mL/min) rates. The Friedman test was used for multivariate analysis between the analyzed time points. For all analyses, a significance level of <5% was considered.

### Ethical aspects

The study was conducted in accordance with the legal and ethical standards recommended by Resolution 466/2012, and was approved by the Research Ethics Committee of the responsible institution under protocol CAEE 31543020.3.0000.5393, on July 8, 2020.

## Results

The results presented were from 203 individuals at high risk of acquiring HIV using daily PrEP and followed for 12 months. Regarding the sociodemographic variables shown in Table 1, 158 (77.8%) of the participants were from the Central SCS, with the largest age group concentrated between 20 and 39 years old, and 158 (77.8%) had a mean age of 34.7±7.7 years. The majority were male at birth (185 (91.1%), of whom 175 (86.2%) were cisgender men. Regarding sexual orientation, the predominant group of participants were homosexual, with 161 (79.3%) people.

Regarding skin color, 134 (66.0%) self-identified as white, followed by 38 (18.7%) as mixed race. Only one (0.5%) participant was homeless. Regarding education, 133 (65.5%) had more than 12 years of study, followed by 8 to 11 years of study, 31 (15.3%) (Table 1).

**Table 1-:** Sociodemographic and clinical profile of people at high risk of acquiring HIV using pre-exposure prophylaxis (n = 203). Ribeirão Preto, SP, Brazil, 2022

**Variables**	**n (%)**
**Health district**	
Central	158 (77.8)
East	20 (9.9)
South	14 (6.9)
North	11 (5.4)
**Age range**	
20-39 years old	158 (77.8)
40-49 years old	37 (18.3)
≥50 years old	8 (3.9)
**Sex at birth**	
Female	18 (8.9)
Male	185 (91.1)
**Gender identity**	
Cis woman	18 (8.9)
Cis man	175 (86.2)
Trans woman	6 (2.9)
Transvestite	4 (2.0)
**Sexual orientation**	
Heterosexual	25 (12.3)
Homosexual	161 (79.3)
Bisexual	17 (8.4)
**Skin color**	
Ignored	10 (4.9)
White	134 (66.0)
Black	19 (9.4)
Brown	38 (18.7)
Yellow	2 (1.0)
**Homelessness**	
Ignored	4 (2.0)
No	198 (97.5)
Yes	1 (0.5)
**Education**	
Unknown/no information	31 (15.3)
No/no formal education	0 (0.0)
1 to 3 years of education	0 (0.0)
4 to 7 years of education	8 (3.9)
8 to 11 years of education	31 (15.3)
More than 12 years of education	133 (65.5)
**SAH** *	
No	199 (98.0)
Yes	4 (2.0)
**Use of antihypertensives**	
No	199 (98.0)
Yes	4 (2.0)
**Dyslipidemia**	
No	201 (99.0)
Yes	2 (1.0)
**Total**	203 (100)

*SAH = Systemic Arterial Hypertension

Regarding renal function assessment, there was a low incidence of kidney injury markers in the participants’ urine tests during the 48 weeks of follow-up. No participant had moderate or severe proteinuria detected throughout the follow-up period. Mild proteinuria (trace or 1+) was observed in a low percentage of participants and remained relatively stable throughout the 48 weeks. A slight increase was observed at week 36, with a return to baseline values at the end of the 48 weeks (Table 2).

**Table 2-:** Monitoring of kidney injury markers according to the time of use of HIV pre-exposure prophylaxis (n = 203). Ribeirão Preto, SP, Brazil, 2022

**Variables**	**Basal n (%)**	**12 weeks n (%)**	**24 weeks n (%)**	**36 weeks n (%)**	**48 weeks n (%)**
**Proteinuria**					
Ignored	22 (10.8)	9 (4.4)	13 (6.4)	12 (5.9)	14 (6.8)
Absent	163 (80.3)	174 (85.7)	177 (87.2)	168 (82.7)	178 (87.7)
Traces*	16 (7.9)	15 (7.4)	10 (4.9)	19 (9.4)	8 (4.0)
1+*	2 (1.0)	5 (2.5)	3 (1.5)	4 (2.0)	3 (1.5)
2+ ^†^	0	0	0	0	0
3+ ^‡^	0	0	0	0	0
4+ ^‡^	0	0	0	0	0
**Glycosuria**					
Ignored	17 (8.4)	9 (4.4)	8 (4.0)	8 (4.0)	10 (4.9)
Missing	185 (91.1)	192 (94.6)	194 (95.5)	194 (95.5)	190 (93.6)
Traces or 1 +*	0	1 (0.5)	0	0	2 (1.0)
2 + ^†^	1 (0.5)	0	0	0	1 (0.5)
3 + ^‡^	0	0	0	0	0
4 + ^‡^	0	1 (0.5)	1 (0.5)	1 (0.5)	0
**Uric acid**					
Ignored	18 (8.9)	10 (4.9)	10 (4.9)	11 (5.4)	10 (4.9)
Absent	185 (91.1)	191 (94.1)	193 (95.1)	192 (94.6)	193 (95.1)
Present	0	2 (1.0)	0	0	0
**Hyaline cylinder**					
Ignored	18 (8.9)	10 (4.9)	10 (4.9)	11 (5.4)	10 (4.9)
Absent	185 (91.1)	192 (94.6)	191 (94.1)	192 (94.6)	192 (94.6)
Present	0	1 (0.5)	2 (1.0)	0	1 (0.5)
**Granular cylinder**					
Ignored	18 (8.9)	10 (4.9)	10 (4.9)	11 (5.4)	10 (4.9)
Absent	185 (91.1)	193 (95.1)	193 (95.1)	192 (94.6)	192 (94.6)
Present	0	0	0	0	1 (0.5)

Proteinuria and Glycosuria: ^*^Trace or 1+ = Grade 1 (mild); ^†^2+= Grade 2 (moderate); ^‡^3+ or 4+=Grade 3 (severe)

Regarding the quarterly assessment of renal function by eGFR (Cockcroft-Gault and CKD-EPI), no significant reduction was observed, remaining normal throughout the time of exposure to TDF. It is worth noting that eGFR by Cockcroft-Gault and CKD-EPI differed considerably from each other from baseline, when a mean of 121.42 ± 33.46 and 100.42 ± 17.71 were observed, respectively. This difference persisted until 48 weeks of follow-up, with a mean of 121.42 ± 33.46 and 101.63 ± 16.69, respectively. Mean serum creatinine remained normal throughout the follow-up period (Table 3).

**Table 3-:** Comparison of serum creatinine, creatinine clearance, eGFR (Cockcroft-Gault)* and eGFR (CKD-EPI)^†^ according to the duration of HIV pre-exposure prophylaxis use (n = 203). Ribeirão Preto, SP, Brazil, 2022

**Variables**	**Mean±SD**	**CI (95%)**	**Min-Max**	**P-value** ^‡^
**Serum Creatinine**				
Basal	1.01±0.19	(0.98-1.04)	(0.50-1.70)	0.244
12 weeks	1.00±0.18	(0.98-1.03)	(0.52-1.80)	
24 weeks	0.98±0.17	0.96-1.01	(0.50-1.50)	
36 weeks	0.99±0.19	0.96-1.01	(0.50-1.60)	
48 weeks	1.00±0.17	0.97-1.02	(0.60-1.53)	
eGFR (CG)*				
Basal	121.42 ± 33.46	116.79-126.05	(64-275)	0.440
12 weeks	120.32 ± 31.19	116.01-124.64	(52-238)	
24 weeks	123.36 ± 32.62	118.85-127.88	(61-265)	
36 weeks	123.17 ± 31.80	118.77-127.57	(62-242)	
48 weeks	121.43 ± 31.44	117.08-125.78	(62-277)	
**eGFR (CKD-EPI)** ^†^				
Basal	100.42±17.71	97.97-102.88	(54-147)	0.510
12 weeks	100.67±16.42	98.39-102.94	(50-148)	
24 weeks	102.59±16.27	100.34-104.84	(64-145)	
36 weeks	102.32±17.70	99.87-104.77	(58-132)	
48 weeks	101.63±16.69	99.32-103.94	(60-130)	

*eGFR = Estimated Glomerular Filtration Rate by Cockcroft-Gault (mL/min); ^†^eGFR = Estimated Glomerular Filtration Rate by CKD-EPI (mL/min/1.73 m^2^); ^‡^P-value = Friedman test, at the 5% significance level

**Figure 1-:**
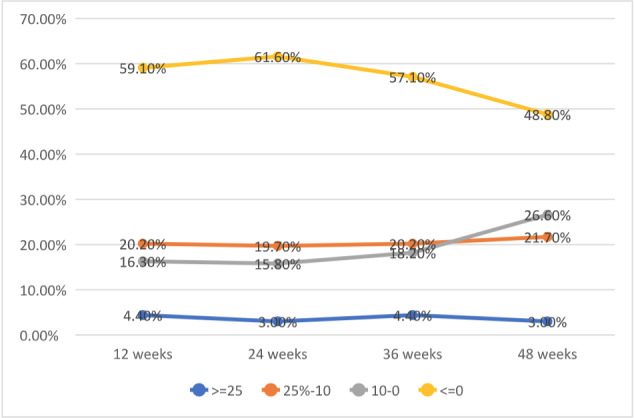
Distribution of reductions in estimated glomerular filtration rate compared to baseline at weeks 12, 24, 36, and 48 of follow-up (n = 203). Ribeirão Preto, SP, Brazil, 2022

Note: The number of participants between follow-up weeks are those in which there was a reduction in renal function

The distribution of participants who had a reduction in eGFR at the four assessment times (12th, 24th, 36th and 48th week) was demonstrated in Figure 1, which shows that the proportion of participants with a decrease in eGFR ≥25% in the 12th and 36th weeks of PrEP use was 4.4%. There was also a fluctuation in improvement in the reduction rate at the 24th and 48th week to 3%.

Table 4 shows the distribution of participants who had an eGFR reduction ≥25% and <25% who had the presence of markers of kidney injury. Therefore, not all participants had an eGFR reduction or the presence of markers of kidney injury. Of the nine participants with an eGFR reduction in the first 12 weeks of PrEP use, 25% (n=2) had proteinuria and an eGFR reduction ≥25%. It is also observed that the majority of PrEP users with proteinuria had an eGFR reduction <25%.

**Table 4-:** Association between reduction in estimated Glomerular Filtration Rate (eGFR) and markers of kidney injury according to duration of HIV pre-exposure prophylaxis use. Ribeirão Preto, SP, Brazil, 2022

**Kidney injury markers**	**Reduction in estimated Glomerular Filtration Rate (eGFR)**											
	**12 weeks**		p*	**24 weeks**		p*	**36 weeks**		p*	**48 weeks**		p*
	≥25%	<25%		≥25%	<25%		≥25%	<25%		≥25%	<25%	
	n (%)	n (%)		n (%)	n (%)		n (%)	n (%)		n (%)	n (%)	
**Proteinuria**												
Absent	6 (75.0)	90 (92.8)	0.084	5 (100)	172 (93.0)	0.539	8 (88.9)	86 (88.7)	0.983	4 (80)	97 (94.2)	0.209
Present **^†^**	2 (25.0)	7 (7.2)		0	13 (7.0)		1 (11.1)	11 (11.3)		1 (20)	6 (5.8)	
**Glycosuria**												
Absent	8 (100)	100 (100)	-	5 (100)	189 (99.5)	0.871	9 (100)	98 (100)	-	5 (100)	102 (99.0)	0.825
Present ^‡^	0	0		0	1 (0.5)		0	0		0	1 (1.0)	
**Uric acid**												
Absent	8 (100)	99 (100)	-	5 (100)	188 (100)	-	9 (100)	97 (100)	-	5 (100)	103 (100)	-
Present	0	0		0	0		0	0		0	0	
**Hyaline cylinder**												
Absent	8 (100)	99 (100)	-	5 (100)	186 (98.9)	0.817	9 (100)	97 (100)	-	5 (100)	102 (99.0)	0.049
Present	0	0		0	2 (1.1)		0	0		0	1 (1)	
**Granular cylinder**												
Absent	8 (100)	99 (100)	-	5 (100)	188 (100)	-	9 (100)	97 (100)	-	5 (100)	102 (99.0)	0.825
Present	0	0		0	0		0	0		0	1 (1)	

*p-value for the Friedman Test; ^†^Present and ^‡^Present: sum of all present results (traces, one cross, two crosses, three crosses and four crosses). The n presented in each marker of kidney injury during the 48 weeks of follow-up is less than the total number of participants, n=203

## Discussion

This study identified a predominance of PrEP users who were male, cisgender, homosexual, White, highly educated, and young. The presence of kidney damage markers capable of predicting the progression of kidney injury was low over a 48-week follow-up period. Furthermore, the results of this study confirm the low incidence of events characterizing renal dysfunction, with the presence of mild and isolated events of proteinuria, glycosuria, uricosuria, and cylindruria in PrEP users. There was no significant association between a decline in eGFR ≥25% and the presence of any of the kidney damage markers studied.

Based on the sociodemographic characteristics of this study, it was possible to identify a predominance of PrEP users who were male at birth and of a young age group (mean age 34 years). These profiles have been evidenced in other studies evaluating the renal function of people using PrEP internationally^(13,19)^. Similarly, a decline in renal function among older individuals who initiated TDF/FTC use was observed in a retrospective cohort study^(24)^.

Regarding monitoring markers of renal damage at 48 weeks, we found that the proportions of proteinuria and glycosuria were low. This finding was also identified in a study of 1,589 men who have sex with men, where 12% had proteinuria and 1% had glycosuria^(25)^. Regarding the first 12 weeks of PrEP use, a prospective cohort study reported a 15% incidence of proteinuria compared to baseline (p<0.0001)^(12)^, diverging from our findings, which showed proteinuria in 2.6% of the population using PrEP. The Partners PrEP study, which performed a sensitivity analysis, observed similar patterns in the distribution of glycosuria between the TDF/FTC group and the placebo group. However, protein excretion and increased urinary uric acid excretion were predominant in those using PrEP^(26)^. Although there was no difference in the risk of proximal tubulopathy, defined as ≥ 2 markers of kidney damage, participants in the TDF/FTC arm had a higher risk of tubular proteinuria compared to placebo^(26)^. Other researchers also suggest that the presence of markers of kidney damage after six months of PrEP use may result in subclinical tubular dysfunction^(27)^. Regarding eGFR, although there was a reduction in its mean calculated by GC and CKD-EPI in our study, the decrease was not significant. This corroborates the multicenter demonstration project PrEP Brazil, which identified a significant reduction in eGFR of -3.46 ± 13.29 (p<0.001) at week 4 compared to baseline. However, there was no statistical difference between the variations in subsequent weeks^(10)^. This profile was also identified in Australia among participants in the EPIC-NSW study, where there was a significant initial decrease (p<0.05) and a gradual and progressive decline. However, there was no record of renal failure over an 18-month observation period^(28)^.

It was also possible to identify that between eGFR calculated by CG and CKD-EPI, there was a wide difference between their means from baseline to week 48: 21; 19.65; 20.78; 20.85 and 19.8 mL/min/1.73 m^2^ at baseline, 12, 24, 36, and 48 weeks, respectively. This difference is also reported in an international study^(12)^.

Regarding the cutoff point of eGFR decline ≥25% from baseline at any time point, in our sample, we identified a small variation in decline among PrEP users at 48 weeks of follow-up. A similar incidence is reported in a study conducted in France and Canada, which reported a ≳20% decline in 4% of PrEP users after six months of PrEP use^(17)^. Meanwhile, in a Brazilian study, 1.8% of PrEP users had an eGFR reduction >30% from baseline^(11)^. Furthermore, it is noteworthy that those who start using PrEP with eGFR <90mL/min/1.73m^2^ have a lower risk of a decline >25%^(28)^.

In our study, we found that a decline in eGFR >25% compared to baseline in individuals using daily oral PrEP was not significantly associated with tubulopathy over 48 weeks, nor did markers of renal damage predict a clinically relevant decline in eGFR. A systematic review of the safety of TDF-based PrEP found that, even though there were no significant differences in eGFR decline, markers of proximal tubular dysfunction individually appeared to be associated with a greater chance of a ≥25% decline in eGFR^(26)^. This suggests that routine monitoring of these markers of proximal tubulopathy is not an efficient approach, as their presence is a rare event. However, it is prudent to perform more frequent assessments in individuals who demonstrate proximal tubulopathy based on the presence of markers of renal damage^(26)^.

Therefore, the recent Therapeutic Guidelines Protocol (PCDT) provides relevant updates. Young individuals without comorbidities will be assessed annually, while those aged 50 or older and those with risk factors for kidney disease, such as DM, hypertension, or a history of kidney disease, and/or a baseline eGFR <90 mL/min, will be monitored biannually. Those without any of these profiles will have their renal function assessed annually^(9)^. This change in the frequency of renal monitoring may have a positive impact on public health management, reducing costs associated with excessive laboratory testing, in addition to promoting greater adherence among users.

The low incidence and lack of association with factors related to renal dysfunction found in our study do not negate the relevance of assessing renal function, as the rates of decline may be mild, but they do exist and are evidenced by TDF nephrotoxicity. Therefore, it is necessary to monitor renal function regularly and periodically, depending on age group and the presence of existing CKD risk factors^(9,28)^. It is reaffirmed, as recent studies have shown, that given the low incidence of eGFR declines of ≥25% compared to baseline eGFR, it is worthwhile considering more frequent renal function monitoring, provided that PrEP users are carefully regrouped based on their personal history of CKD risk factors and age group, and are constantly reassessed for possible health changes that may occur during PrEP use^(10,29)^.

It is worth noting that there is still a scarcity of studies in the literature evaluating long-term eGFR decline in PrEP users, as the longest studies involve follow-up periods of up to three years^(4)^. This is a potential focus for future research, given that the nephrotoxic action of TDF and its effect on the progressive decline in eGFR in people living with HIV have already been scientifically proven^(30)^.

A limitation of this study is the lack of information obtained from medical records regarding family and personal history, and medication use, as such data are relevant and could be related to the risk of eGFR decline. Furthermore, data regarding laboratory test results, which should be recorded in the Hygiaweb electronic system, were also overlooked. Despite this, the data obtained are the same as those used to monitor people using PrEP in clinical practice and allowed for a 48-week follow-up study, as found in other studies evaluating renal function in people using PrEP.

Among the strategies to expand access to the use of PrEP in Brazilian states and municipalities, and achieve the goal of eliminating HIV infection as a public health problem in Brazil, the Ministry of Health included nurses as members of the health team to prescribe PrEP and carry out clinical follow-up of people using PrEP, within the scope of the SUS, according to the Clinical Protocol and Therapeutic Guidelines for Pre-Exposure Prophylaxis to HIV (PCDT/PrEP) as long as they are established in public health programs and in a routine approved by the health institution^(9)^, with the aim of reducing the number of new infections in line with the SDGs^(9)^.

It is worth noting that recognizing nurses’ technical and scientific competence demonstrates their relevance to advancing prevention and care policies in the context of HIV infection and represents an expansion of the scope of clinical nursing practice^(31)^. Nurses should include in all nursing consultations with PrEP users the assessment of renal function markers and implement nursing interventions that involve health literacy regarding the importance of clinical and laboratory follow-up, clarifying the importance of renal function assessment, especially among those at higher risk of alterations^(31-32)^, since individuals’ sociodemographic and clinical characteristics are important for implementing appropriate personalized health literacy strategies^(33)^.

It is important to emphasize the importance of clinical nursing assessment in the context of PrEP prescription and comprehensive monitoring of people using PrEP, including ordering tests for adequate renal function monitoring. Nurses’ clinical knowledge of renal function monitoring is essential for the early identification of risk factors and the careful management of PrEP inclusion, maintenance, and eventual discontinuation processes, ensuring the appropriate and safe indication of this prevention strategy, considering each individual’s clinical uniqueness^(34)^.

The renal function monitoring of individuals using PrEP, as proposed in this study, makes a significant contribution to the advancement of scientific knowledge, given that there are still few studies focusing on the effects of PrEP on markers of renal function over time, particularly at the national level. This contributes to consensus on the use of this important prevention strategy. This knowledge reinforces the recommendations of the Brazilian protocol and highlights the importance of health and nursing interventions focused on monitoring and self-care, particularly among individuals using PrEP who have risk factors for renal dysfunction.

## Conclusion

People using PrEP who were followed for 48 weeks were mostly men, cisgender, homosexual, white, highly educated, and young with a mean age of 34 years. The presence of markers of kidney damage predicts low kidney injury in people using PrEP. Among the markers, proteinuria increased until the 12th week (2.6%), maintaining a small variation until the 48th week. All cases were mild or grade 1 proteinuria events. Glycosuria was even rarer; however, one participant’s condition progressed from grade 2 to grade 4.

In our study, we did not identify changes in mean serum creatinine levels during the follow-up weeks. However, eGFR calculated by Cockcroft-Gault showed a slight and progressive decline at weeks 12, 36, and 48, while CKD-EPI decreased only at weeks 36 and 48. The eGFR calculated by CG compared with CKD-EPI remained approximately 20 mL/min apart throughout the study period, with eGFR calculated by CG being higher than that calculated by CKD-EPI.

Furthermore, the results of this study confirm the low incidence of events characterizing renal dysfunction. During the 48 weeks of study follow-up, between 3% and 4.4% of the population experienced a decline in eGFR equal to or greater than 25%, in addition to mild and isolated events of proteinuria, glycosuria, uricosuria, and cylindruria in people using PrEP. There was no significant association between a decline in eGFR ≥25% and the presence of any of the markers of kidney damage studied.

## Data Availability

Datasets related to this article will be available upon request to the corresponding author.
