# Supplementary material for: Effect of antiretrovirals on renal function in people taking HIV pre-exposure prophylaxis
Source: Rev Lat Am Enfermagem. 2025 Nov 17;33:e4734. doi: 10.1590/1518-8345.7280.4734 (PMC12628367; doi:10.1590/1518-8345.7280.4734)
Supplement: Translation

# Translation: Efeito dos antirretrovirais na função renal das pessoas em uso da profilaxia pré-exposição ao HIV*

**DOI:** 10.1590/1518-8345.7280.4735

**Authors:** Priscila Silva Pontes-Pereira, Rodrigo de Carvalho Santana, Elucir Gir, Renata Karina Reis

**Affiliations:** 1 Universidade de São Paulo, Escola de Enfermagem de Ribeirão Preto, Centro Colaborador da OPAS/OMS para o Desenvolvimento da Pesquisa em Enfermagem, Ribeirão Preto, SP, Brasil.; 2 Universidade de São Paulo, Faculdade de Medicina de Ribeirão Preto, Ribeirão Preto, SP, Brasil.

**Keywords:** Profilaxia Pré-Exposição, Antirretrovirais, Tenofovir, Nefropatias, Monitorização Fisiológica, Taxa de Filtração Glomerular

## Abstract

avaliar o efeito dos antirretrovirais na função renal das pessoas em uso da profilaxia pré-exposição ao HIV.

delineamento descritivo tipo levantamento longitudinal, feito na coorte retrospectiva por 48 semanas. A amostra foi de 203 participantes. A amostragem foi não probabilística por conveniência. Analisou-se variáveis sociodemográficas, clínicas e laboratoriais. Utilizou-se frequência absoluta, relativa, média, desvio-padrão, mínima e máxima, teste exato de Fisher, teste Friedman, nível de significância < 5%.

predominantemente homens cis (86,2%), cor de pele branca (69,1%), alta escolaridade (76,7%) e idade média de ± 34 anos. A presença de marcadores de dano renal foi baixa. A proteinúria teve um aumento até a 12ª semana (2,6%), mantendo uma pequena variação até 48ª semana. O declínio da estimativa da Taxa de Filtração Glomerular (eTFG) ≥ 25% em relação aos níveis basais foi de 4,4%, 3%, 4,4% e 3% nas 12ª, 24ª, 36ª e 48ª semanas respectivamente. Não houve associação significativa entre declínio da eTFG ≥ 25% e presença de um dos marcadores de dano renal.

ratifica-se a baixa incidência de eventos que caracterizam disfunção renal, como o declínio da eTFG ≥ 25% e a presença de eventos leves e isolados de proteinúria, glicosúria, uricosúria e cilindrúria em 48 semanas de acompanhamento.

## Destaques

**(1)** Poucos estudos sobre disfunção renal em usuários da profilaxia pré-exposição ao HIV. **(2)** Baixa incidência de disfunção renal em usuários da PrEP durante 48 semanas. **(3)** Baixa incidência de marcadores de dano renal durante 48 semanas de acompanhamento. **(4)** O conhecimento clínico do enfermeiro para o monitoramento da função renal é essencial.

## Introdução

O enfraquecimento do progresso da resposta ao Vírus da Imunodeficiência Humana (HIV) nos últimos anos reflete uma preocupante reemergência da epidemia da infecção pelo HIV^(1)^. Os relatórios globais apontam um total de 1,3 milhão de novas infecções por HIV em 2023^(2)^ e, se tais tendências se mantiverem, 1,2 milhão de pessoas serão infectadas pelo vírus em 2025, ou seja, três vezes mais do que a meta para este ano^(3)^.

De acordo com as metas propostas pelas Nações Unidas para o alcance dos Objetivos de Desenvolvimento Sustentável (ODS), busca-se reduzir a incidência de novos casos do HIV no mundo para um total de 200.000 e eliminar a epidemia da aids como problema de saúde pública até 2030^(2)^. Nesse contexto, observa-se mudança no paradigma da prevenção da transmissão sexual do HIV decorrente dos avanços obtidos nos últimos anos com a proposta da prevenção combinada, a qual envolve a recomendação e disponibilização de diversas estratégias, com destaque para as intervenções biomédicas baseadas no uso de medicamentos antirretrovirais, como a Profilaxia Pré-Exposição ao HIV (PrEP), considerada um dos mais importantes avanços recentes na prevenção da infecção pelo HIV^(4)^. A PrEP se baseia na combinação de antirretrovirais, sendo uma das mais comuns a associação de Tenofovir Disoproxil Fumarato (TDF) e Entricitabina (FTC) oral (TDF/FTC) em dose fixa 300/200 mg^(4)^.

Apesar dos diversos benefícios do uso regular da PrEP, as pessoas que a utilizam devem ser monitoradas quanto aos potenciais efeitos adversos do seu uso constante e regular^(5)^. Vale ressaltar que já é estabelecido na literatura que o TDF está associado à lesão tubular renal proximal, obstrução induzida por cristais ou nefrite intersticial^(6)^, necrose tubular aguda tóxica com dismorfia e alargamento mitocondrial distinto^(7)^. Ensaios clínicos mostraram que o mesmo está associado ao aumento do risco de Insuficiência Renal Aguda (IRA), proteinúria nefrogênica, Diabetes Insípido, síndrome de Fanconi e Doença Renal Crônica (DRC)^(8-9)^.

Dessa forma, a preocupação com a disfunção renal entre pessoas que fazem uso da PrEP é relevante para a prática clínica. Diferentes estudos descrevem declínio da estimativa da Taxa de Filtração Glomerular (eTFG) pelo TDF em pessoas usuárias de PrEP, variando de 1% a 15%^(10-14)^. Essa ampla variabilidade pode estar atrelada à decorrência de diferentes conjunturas populacionais investigadas, incluindo sexo, etnia e idade^(10-14)^. Outros indicadores de lesão renal são relatados, como o declínio da eTFG ≥ 25% em relação à taxa basal^(15)^; presença de marcadores de tubulopatia proximal como creatininúria, proteinúria não albumina, glicosúria euglicêmica, aumento da excreção urinária de fosfato, aumento da excreção urinária de ácido úrico^(15-17)^.

A avaliação cuidadosa dos efeitos adversos em nível renal é essencial para maximizar os benefícios e minimizar os riscos^(9)^. O acompanhamento rotineiro da função renal é crítico em pessoas que recebem TDF, visto que o uso contínuo de uma droga antirretroviral nefrotóxica será incluso no cotidiano dessas pessoas como um método profilático para a prevenção do HIV^(9)^.

Diante da temática exposta e da comprovada relevância internacional da pauta relacionada à ação de um antirretroviral nefrotóxico utilizado como agente profilático de prevenção ao HIV, desde indicação do método preventivo à frequência de avaliação, impactos na adesão medicamentosa, clínicos e econômicos justificam, portanto, o atual interesse entre os cientistas e enfermeiros clínicos para chegar a consensos sobre o uso da PrEP a nível renal. Portanto, o objetivo do estudo apresentado foi avaliar o efeito dos antirretrovirais na função renal das pessoas em uso da PrEP.

## Método

### Delineamento do estudo

Trata-se de um estudo do tipo coorte retrospectivo realizado em pessoas soronegativas ao HIV em uso da PrEP por 48 semanas. O período de 48 semanas de acompanhamento das pessoas em uso da PrEP correspondeu aos que iniciaram o seu uso entre janeiro de 2018 a janeiro de 2021. A coleta de dados transcorreu em um período de quatro meses, compreendidos entre maio e agosto de 2022.

### Local da coleta de dados

Os participantes elegíveis foram identificados dentre as pessoas usuárias de quatro Serviços de Atendimento Especializado (SAE) correspondendo à região central, leste, sul e norte localizados no sudeste do Brasil, especificamente em um município no interior do estado de São Paulo.

### População

Inicialmente foram identificados, nos SAE, 614 pessoas que iniciaram o uso da PrEP entre janeiro de 2018 a janeiro de 2021, sendo distribuídos nos SAE da região central (419), leste (52), sul (61) e norte (64).

### Critério de seleção

Dentre os critérios de seleção deste estudo, foram considerados os seguintes de inclusão: pessoas em uso da PrEP com idade ≥ 18 anos; disponibilidade dos registros em prontuário de, pelo menos, uma consulta de retorno e quatro de acompanhamento trimestrais, bem como os resultados dos exames laboratoriais; início da PrEP entre janeiro de 2018 a janeiro de 2021; função renal normal (creatinina sérica ≤1,3 mg/dL para homens / ≤1,1 mg/dL para mulheres e eTFG ≥60 mL/min) antes de iniciar a PrEP. Os critérios de exclusão foram: não ter completado o período de seguimento regular de 48 semanas; ausência de registro completo das informações e resultados laboratoriais em prontuário dos participantes e ter diagnóstico de diabetes mellitus (DM).

### Definição da amostra

A amostra foi não probabilística, consecutiva, composta por todas as pessoas usuárias da PrEP que iniciaram o seu uso entre janeiro de 2018 a janeiro de 2021. O tamanho da amostra incluiu a população elegível no período de coleta de dados, ou seja, todos os registros dos prontuários das pessoas em uso da PrEP foram consultados e avaliados quanto aos critérios de elegibilidade do estudo de forma manual, não probabilística, resultando em um total de 203 participantes, pertencentes aos SAE das regiões central (158), leste (20), sul (14) e norte (11). Vale ressaltar a ocorrência de dados ignorados nos prontuários dos participantes ao longo das 48 semanas de acompanhamento.

### Variáveis do estudo

As variáveis consideradas para este estudo foram as sociodemográficas, clínicas gerais e laboratoriais.

As variáveis sociodemográficas compreendiam: sexo de nascimento (feminino, masculino), orientação sexual (heterossexual, homossexual, bissexual, ignorado); identidade de gênero (homem cisgênero, homem transgênero, mulher cisgênero, mulher transgênero, travesti, pansexual, outros); idade em anos completos; faixa etária (20-39 anos / 40-49 anos / ≥ 50 anos); cor de pele autorreferida (branco, preto, amarelo, pardo, não declara); escolaridade (nenhum / sem educação formal / 1 a 3 anos de estudo / 4 a 7 anos de estudo / 8 a 11 anos de estudo / ≥ 12 anos de estudo); situação de rua (sim, não).

Em relação às variáveis clínicas gerais e laboratoriais, considerou-se para o estudo: histórico de hipertensão arterial sistêmica (HAS) (sim, não); dislipidemia (sim, não); uso de medicamentos anti-hipertensivos (sim, não); dosagem de creatinina sérica (mg/dL); eTFG; exame de urina para avaliação de proteinúria [ausente, presente-traços, uma cruz (1+), duas cruzes (2+), três cruzes ou mais (3+)], glicosúria [(ausente, presente- (1+), duas cruzes (2+), três cruzes ou mais (3+)], ácido úrico (presente, ausente), cilindros hialinos (presente, ausente) e cilindros granulosos (presente, ausente).

### Instrumentos utilizados para a coleta das informações

Para a coleta de dados, utilizou-se um questionário semiestruturado submetido à validação teórica de face e conteúdo por cinco expertises da área, que o avaliaram quanto à pertinência, fácil compreensão e relevância das variáveis selecionadas.

Tanto o questionário de coleta de dados como o questionário de validação foram enviados por e-mail para cinco juízes especialistas em infectologia, nefrologia e metodologia, os quais foram identificados por meio de currículo Lattes, cujo prazo foi estipulado em 15 dias. Após a análise e ajustes necessários, fez-se um estudo-piloto com 15 prontuários de pessoas em uso da PrEP em um dos SAE. Tal instrumento foi utilizado especificamente visando o alcance dos objetivos deste estudo. Vale destacar que os 15 prontuários do teste-piloto não foram incluídos na amostra final do estudo.

Para a avaliação da função renal, utilizou-se a eTFG calculada pelas equações matemáticas empíricas baseadas na dosagem de creatinina sérica: *Chronic Kidney Disease Epidemiology Collaboration* (CKD-EPI) (mL/min/1,73 m^2^)^(18)^ e Cockcroft-Gault (CG) (mL/min)^(19)^, o qual foi utilizado como instrumento a calculadora no aplicativo da *National Kidney Foundation* (NKF)^(20)^.

Para avaliar a escala de gravidade dos eventos adversos, especificamente os marcadores de dano renal (proteinúria, glicosúria e cilindrúria), utilizou-se a escala de gravidade dos eventos adversos em adultos usados pela *France Recherche Nord et Sud Sida-HIV et Hepatites* [Agência Nacional de Pesquisa em AIDS e Hepatite (ANRS)], o qual gradua os eventos em: Grau 1 ou evento leve (traços a 1+); grau 2 ou evento moderado (2+) e grau 3 ou evento severo (3+ ou mais)^(21)^.

Para identificar o declínio da função renal, utilizamos o acrônimo de RIFLE: *risk* (R: risco), *injury* (I: injúria), *failure* (F: falência), *loss* (L: perda mantida da função) e *end-stage kidney disease* (E: insuficiência renal terminal). O RIFLE estabelece que as três primeiras classes mais sensíveis e referentes a graus de gravidade da disfunção renal são avaliadas por mudanças relativas no valor do nível sérico da creatinina ou na TFG a partir de um valor basal^(22)^. Como marcador de lesão renal clinicamente significativa foi considerado como ponto de corte um declínio significativo da eTFG≥25% em relação aos níveis basais em qualquer ponto de tempo^(22)^.

### Coleta de dados

Os dados foram coletados pela pesquisadora e uma auxiliar de pesquisa após treinamento teórico e prático. A coleta de dados foi seccionada em cinco momentos: linha de base (antes de iniciar a PrEP) e quatro acompanhamentos clínico e laboratoriais trimestralmente totalizando um período de acompanhamento de 48 semanas.

Foram obtidas informações sobre dados sociodemográficos, clínicos gerais e exames laboratoriais, bem como calculou-se a eTFG: pela equação CKD-EPI e CG.

Os dados foram coletados por meio do: formulário de cadastramento de usuário à PrEP, ficha de primeiro atendimento, ficha de retorno de 30 dias, ficha de acompanhamento clínico em PrEP e os exames laboratoriais trimestrais registrados no sistema *Hygiaweb*, um *software* de gestão em saúde utilizado no município onde os SAE estão situados, desenvolvido para automatizar processos e promover a integração entre as unidades de saúde da rede pública, cujas funções incluem a disponibilização de resultado *online* de exames laboratoriais realizados nos serviços de saúde.

Para obter-se as eTFG pelas equações CKD-EPI e CG, foram necessárias informações sobre sexo, idade e creatinina sérica; e sexo, idade, peso e creatinina sérica respectivamente. Para a identificação dos marcadores de dano renal avaliaram-se os resultados de amostra única de urina coletada pela manhã, a qual era analisada trimestralmente, como: os parâmetros químicos: ácido úrico e os biomarcardores urinários: proteinúria e glicosúria (cruzes) e cilindrúria - cilindros hialinos e cilindros granulosos (presente e ausente). Em nosso estudo a proteinúria identificada em exames laboratoriais foi a qualitativa, classificada em cruzes (traços, 1+, 2+, 3+ e 4+).

### Tratamento e análise dos dados:

Os dados do estudo foram coletados e gerenciados usando ferramentas de captura de dados eletrônicos *Research Electronic Data Capture* (REDCap) hospedada e disponibilizada pela instituição proponente^(23)^. O REDCap é uma plataforma de *software* segura e baseada na *web* projetada para suportar a captura de dados para estudos de pesquisa, fornecendo uma interface intuitiva para captura de dados validados e trilhas de auditoria para rastrear manipulação de dados e procedimentos^(23)^.

Os dados foram processados e analisados por meio do *softwareStatistical Package for the Social Sciences* (SPSS), versão 26.0. Utilizou-se estatísticas descritivas por meio de frequência absoluta (n) e relativa (%), média, desvio-padrão (DP), mínima e máxima, e o teste de associação exato de Fisher. Para as variáveis significativas empregou a regressão logística para calcular a razão de chance, *Odds Ratio* (OR). O teste exato de Fisher também foi adotado para mensurar a associação entre redução da função renal e os marcadores de dano renal. O teste de normalidade Kolmogorov-Smirnov^.^ foi aplicado para mensurar a simetria entre a taxas do CKD-EPI (mL/min/1,73 m^2^) e Cockcroft-Gault (mL/min). O teste Friedman foi usado para análise multivariada entre os momentos analisados. Para todas as análises, foi considerado nível de significância <5%.

### Aspectos éticos

O estudo foi conduzido de acordo com as normas legais e éticas recomendadas pela Resolução 466/2012, sendo aprovado pelo Comitê de Ética em Pesquisa da instituição responsável sob o protocolo CAEE 31543020.3.0000.5393, em 08/07/2020.

## Resultados

Os resultados apresentados foram referentes a 203 pessoas sob alto risco de adquirir o HIV em uso da PrEP diária acompanhadas por 12 meses. Em relação às variáveis sociodemográficas demonstradas na Tabela 1, verificou-se que 158 (77,8%) dos participantes eram provenientes do SAE Central, sendo a maior faixa etária concentrada entre 20 e 39 anos de idade e média de idade de 34,7±7,7 anos. A maioria pertencia ao sexo de nascimento masculino 185 (91,1%), destes, 175 (86,2%) eram homens cisgênero. Quanto à orientação sexual, a predominância dos participantes era homossexual com 161 (79,3%) pessoas.

Em relação à cor de pele, 134 (66,0%) se autodeclaravam brancos, seguidos de pardos, 38 (18,7%). Apenas um (0,5%) participante estava em situação de rua. Quanto à escolaridade, 133 (65,5%) tinham mais de 12 anos de estudo, seguido de 8 a 11 anos de estudo, 31 (15,3%) (Tabela 1).

**Tabela 1-:** Perfil sociodemográfico e clínico de pessoas sob alto risco de adquirir o HIV em uso da profilaxia pré-exposição (n = 203). Ribeirão Preto, SP, Brasil, 2022

**Variáveis**	**n (%)**
**Distrito de saúde**	
Central	158 (77,8)
Leste	20 (9,9)
Sul	14 (6,9)
Norte	11 (5,4)
**Faixa etária**	
20-39 anos	158 (77,8)
40-49 anos	37 (18,3)
≥50 anos	8 (3,9)
**Sexo de nascimento**	
Feminino	18 (8,9)
Masculino	185 (91,1)
**Identidade de gênero**	
Mulher cis	18 (8,9)
Homem cis	175 (86,2)
Mulher trans	6 (2,9)
Travesti	4 (2,0)
**Orientação sexual**	
Heterossexual	25 (12,3)
Homossexual	161 (79,3)
Bissexual	17 (8,4)
**Cor de pele**	
Ignorada	10 (4,9)
Branco	134 (66,0)
Preto	19 (9,4)
Pardo	38 (18,7)
Amarelo	2 (1,0)
**Situação de rua**	
Ignorada	4 (2,0)
Não	198 (97,5)
Sim	1 (0,5)
**Escolaridade**	
Ignorado/sem informação	31 (15,3)
nenhuma/sem educação formal	0 (0,0)
1 a 3 anos de estudo	0 (0,0)
4 a 7 anos de estudo	8 (3,9)
8 a 11 anos de estudo	31 (15,3)
mais de 12 anos de estudo	133 (65,5)
**HAS** *	
Não	199 (98,0)
Sim	4 (2,0)
**Uso de anti-hipertensivo**	
Não	199 (98,0)
Sim	4 (2,0)
**Dislipidemia**	
Não	201 (99,0)
Sim	2 (1,0)
**Total**	203 (100)

*HAS = Hipertensão Arterial Sistêmica

Quanto à avaliação da função renal, houve baixa incidência dos marcadores de lesão renal no exame de urina dos participantes durante as 48 semanas de acompanhamento. Em nenhum participante foi detectada proteinúria moderada ou grave durante todo o seguimento. A presença de proteinúria leve (traços ou 1+) foi observada em baixo percentual de participantes e permaneceu relativamente estável ao longo das 48 semanas. Observou-se uma discreta elevação na semana 36 com retorno aos valores basais ao final das 48 semanas (Tabela 2).

**Tabela 2-:** Monitoramento dos marcadores de lesão renal de acordo com o tempo do uso da profilaxia pré-exposição ao HIV (n = 203). Ribeirão Preto, SP, Brasil, 2022

**Variáveis**	**Basal**	**12 semanas n (%)**	**24 semanas n (%)**	**36 semanas n (%)**	**48 semanas n (%)**
**Proteinúria**					
Ignorado	22(10,8)	9 (4,4)	13 (6,4)	12 (5,9)	14 (6,8)
Ausente	163 (80,3)	174 (85,7)	177 (87,2)	168 (82,7)	178 (87,7)
Traços*	16 (7,9)	15 (7,4)	10 (4,9)	19 (9,4)	8 (4,0)
1+*	2 (1,0)	5 (2,5)	3 (1,5)	4 (2,0)	3 (1,5)
2+ ^†^	0	0	0	0	0
3+ ^‡^	0	0	0	0	0
4+ ^‡^	0	0	0	0	0
**Glicosúria**					
Ignorado	17 (8,4)	9 (4,4)	8 (4,0)	8 (4,0)	10 (4,9)
Ausente	185 (91,1)	192 (94,6)	194 (95,5)	194 (95,5)	190 (93,6)
Traços ou 1 +*	0	1 (0,5)	0	0	2 (1,0)
2 + ^†^	1 (0,5)	0	0	0	1 (0,5)
3 + ^‡^	0	0	0	0	0
4 + ^‡^	0	1 (0,5)	1 (0,5)	1 (0,5)	0
**Ácido úrico**					
Ignorado	18 (8,9)	10 (4,9)	10 (4,9)	11 (5,4)	10 (4,9)
Ausente	185 (91,1)	191 (94,1)	193 (95,1)	192 (94,6)	193 (95,1)
Presente	0	2(1,0)	0	0	0
**Cilindro hialino**					
Ignorado	18 (8,9)	10 (4,9)	10 (4,9)	11 (5,4)	10 (4,9)
Ausente	185 (91,1)	192 (94,6)	191 (94,1)	192 (94,6)	192 (94,6)
Presente	0	1 (0,5)	2 (1,0)	0	1 (0,5)
**Cilindro granuloso**					
Ignorado	18 (8,9)	10 (4,9)	10 (4,9)	11 (5,4)	10 (4,9)
Ausente	185 (91,1)	193 (95,1)	193 (95,1)	192 (94,6)	192 (94,6)
Presente	0	0	0	0	1 (0,5)

Proteinúria e Glicosúria: *Traços ou 1+ = Grau 1 (leve); ^†^2+= Grau 2 (moderado); ^‡^3+ou 4+=Grau 3 (grave)

Quanto à avaliação trimestral da função renal pela eTFG (Cockcroft-Gault e CKD-EPI), não se observou redução significativa, tendo permanecida normal ao longo do tempo de exposição ao TDF. Vale ressaltar que a eTFG por Cockcroft-Gault e CKD-EPI diferiram consideravelmente entre si desde o momento basal quando se observou uma média de 121,42±33,46 e 100,42±17,71, respectivamente. A diferença perdurou até as 48 semanas de acompanhamento com média de 121,42±33,46 e 101,63±16,69, respectivamente. A creatinina sérica média permaneceu normal durante o período de acompanhamento (Tabela 3).

**Tabela 3-:** Comparação da creatinina sérica, *Clearance* de creatinina, eTFG (Cockcroft-Gault)* e eTFG (CKD-EPI)**^†^** de acordo com o tempo do uso da profilaxia pré-exposição ao HIV (n = 203). Ribeirão Preto, SP, Brasil, 2022

**Variáveis**	**Média±Dp** ^‡^	**IC (95%)**	**Min-Máx**	**P-valor** ^§^
**Creatinina sérica**				
Basal	1,01±0,19	(0,98-1,04)	(0,50-1,70)	0,244
12 semanas	1,00±0,18	(0,98-1,03)	(0,52-1,80)	
24 semanas	0,98±0,17	0,96-1,01	(0,50-1,50)	
36 semanas	0,99±0,19	0,96-1,01	(0,50-1,60)	
48 semanas	1,00±0,17	0,97-1,02	(0,60-1,53)	
eTFG (CG)*				
Basal	121,42 ± 33,46	116,79-126,05	(64-275)	0,440
12 semanas	120,32 ± 31,19	116,01-124,64	(52-238)	
24 semanas	123,36 ± 32,62	118,85-127,88	(61-265)	
36 semanas	123,17 ± 31,80	118,77-127,57	(62-242)	
48 semanas	121,43 ± 31,44	117,08-125,78	(62-277)	
**eTFG(CKD-EPI)** ^†^				
Basal	100,42±17,71	97,97-102,88	(54-147)	0,510
12 semanas	100,67±16,42	98,39-102,94	(50-148)	
24 semanas	102,59±16,27	100,34-104,84	(64-145)	
36 semanas	102,32±17,70	99,87-104,77	(58-132)	
48 semanas	101,63±16,69	99,32-103,94	(60-130)	

*eTFG = Estimativa da Taxa de Filtração Glomerular por Cockcroft-Gault (mL/min); ^†^eTFG = Estimativa da Taxa de Filtração Glomerular por CKD-EPI (mL/min/1,73 m^2^); ^‡^Dp = Desvio-Padrão; ^§^P-valor = Teste Friedman, ao nível de significância de 5%

A distribuição dos participantes que tiveram redução da eTFG nos quatro momentos de avaliação (12ª, 24ª, 36ª e 48ª semana) foi demonstrada na Figura 1, a qual aponta que a proporção dos participantes com diminuição da eTFG ≥ 25% nas semanas 12ª e 36ª de uso da PrEP foi de 4,4%. Houve também uma oscilação de melhora na taxa de redução na 24ª e 48ª semana para 3%.

**Figura 1-:** Distribuição das reduções da estimativa da taxa de filtração glomerular em relação à avaliação basal na 12ª, 24ª, 36ª e 48ª semana de acompanhamento (n = 203). Ribeirão Preto, SP, Brasil, 2022

Nota: O n de participantes entre as semanas de acompanhamento são aqueles em que houve redução da função renal

A Tabela 4 mostra a distribuição dos participantes que obtiveram redução da eTFG ≥ 25% e < 25% que tiveram a presença de marcadores de lesão renal, portanto nem todos os participantes tiveram redução de eTFG nem presença de marcadores de lesão renal. Dos nove participantes com redução da eTFG nas primeiras 12 semanas de uso da PrEP, 25% (n = 2) possuíam presença de proteinúria e redução da eTFG ≥ 25%. Observa-se também que a maioria das pessoas usuárias da PrEP com proteinúria presente tiveram redução da eTFG < 25%.

**Tabela 4-:** Associação entre a redução da estimativa da Taxa de Filtração Glomerular (eTFG) e marcadores de lesão renal de acordo com o tempo do uso da profilaxia pré-exposição ao HIV. Ribeirão Preto, SP, Brasil, 2022

**Marcadores de lesão renal**	**Redução da estimativa da Taxa de Filtração Glomerular (eTFG)**											
	**12 semanas**		p*	**24 semanas**		p*	**36 semanas**		p*	**48 semanas**		p*
	≥25% n (%)	<25% n (%)		≥25% n (%)	<25% n (%)		≥25% n (%)	<25% n (%)		≥25% n (%)	<25% n (%)	
**Proteinúria**												
Ausente	6 (75,0)	90 (92,8)	0,084	5 (100)	172 (93,0)	0,539	8 (88,9)	86 (88,7)	0,983	4 (80)	97 (94,2)	0,209
Presente ^†^	2 (25,0)	7 (7,2)		0	13 (7,0)		1 (11,1)	11 (11,3)		1 (20)	6 (5,8)	
**Glicosúria**												
Ausente	8 (100)	100 (100)	-	5 (100)	189 (99,5)	0,871	9 (100)	98 (100)	-	5 (100)	102 (99,0)	0,825
Presente ^‡^	0	0		0	1 (0,5)		0	0		0	1 (1,0)	
**Ácido úrico**												
Ausente	8 (100)	99 (100)	-	5 (100)	188 (100)	-	9 (100)	97 (100)	-	5 (100)	103 (100)	-
Presente	0	0		0	0		0	0		0	0	
**Cilindro hialino**												
Ausente	8 (100)	99 (100)	-	5 (100)	186 (98,9)	0,817	9 (100)	97 (100)	-	5 (100)	102 (99,0)	0,049
Presente	0	0		0	2 (1,1)		0	0		0	1 (1)	
**Cilindro granuloso**												
Ausente	8 (100)	99 (100)	-	5 (100)	188 (100)	-	9 (100)	97 (100)	-	5 (100)	102 (99,0)	0,825
Presente	0	0		0	0		0	0		0	1 (1)	

*p-valor para o Teste Friedman; ^†^Presente e ^‡^Presente: somatório de todos os resultados presentes (traços, uma cruz, duas cruzes, três cruzes e quatro cruzes. O n apresentado em cada marcador de lesão renal durante as 48 semanas de acompanhamento é menor que o número total de participantes, n=203

## Discussão

Neste estudo, foi possível identificar uma predominância de pessoas usuárias da PrEP do sexo masculino, cis, homossexual, branco, com alta escolaridade e jovens. A presença de marcadores de dano renal capazes de predizer a evolução de lesão renal foi baixa em um período de 48 semanas de acompanhamento. Além disso, os resultados da pesquisa ratificam a baixa incidência de eventos que caracterizam disfunção renal com presença de eventos leves e isolados de proteinúria, glicosúria, uricosúria e cilindrúria em pessoas usuárias de PrEP. Não houve associação significativa entre o declínio da eTFG ≥ 25% e presença de um dos marcadores de dano renal estudados.

A partir da caracterização sociodemográfica deste estudo, foi possível identificar uma predominância de pessoas usuárias da PrEP do sexo de nascimento masculino e faixa etária jovem com uma média de 34 anos. Tais perfis foram evidenciados em outros estudos que avaliaram a função renal das pessoas em uso da PrEP em âmbito internacional^(13,19)^. Além disso, o declínio da função renal entre as pessoas com idade mais avançada que iniciaram o uso da TDF/FTC foi observada em uma coorte retrospectiva^(24)^.

Em relação ao monitoramento dos marcadores de dano renal em 48 semanas, identificamos que a proporção da proteinúria e glicosúria foram baixas. Tal achado é identificado também em um estudo realizado com 1.589 homens que fazem sexo com homens, onde 12% apresentaram proteinúria e 1% glicosúria^(25)^. Em relação às primeiras 12 semanas de uso da PrEP, uma coorte prospectiva apresentou uma incidência de 15% de proteinúria em relação à linha de base (p<0,0001)^(12)^, divergindo dos nossos achados, no qual apresentou-se proteinúria em 2,6% da população em uso da PrEP.

O estudo *Partners PrEP* que realizou uma análise de sensibilidade observou padrões semelhantes na distribuição de glicosúria entre o grupo TDF/FTC e o grupo placebo, entretanto a excreção de proteína e aumento da excreção do ácido úrico na urina eram predominantes nas pessoas em uso da PrEP^(26)^. Embora não houvesse diferença no risco de tubulopatia proximal, definida como ≥ 2 indicadores de dano renal, os participantes do braço TDF/FTC tiveram um risco maior de proteinúria tubular em comparação com o placebo^(26)^. Outros pesquisadores sugerem também que a presença de marcadores de dano renal após seis meses de uso da PrEP pode resultar em disfunção subclínica do túbulo^(27)^.

Em relação à eTFG, apesar de haver uma redução de sua média calculada por CG e CKD-EPI em nosso estudo, o decréscimo não foi significante; corroborando com o projeto demonstrativo multicêntrico - PrEP Brasil, no qual se identificou uma redução significativa na eTFG de − 3,46 ± 13,29 (p<0,001) na semana 4 em relação à avaliação basal, entretanto não houve diferença estatística entre as variações nas semanas subsequentes^(10)^. Este perfil também foi identificado na Austrália em participantes do estudo EPIC-NSW, no qual houve uma queda inicial significativa (p<0,05) e um declínio gradual e progressivo, porém não houve registro de insuficiência renal em um período de 18 meses de observação^(28)^.

Também foi possível identificar que entre a eTFG calculada por CG e CKD-EPI houve uma ampla diferença entre suas médias desde a linha de base até a 48ª semana de 21; 19,65; 20,78; 20,85 e 19,8 mL/min/1,73 m^2^ nos momentos basal, 12ª, 24ª, 36ª e 48ª semanas, respectivamente. Esta diferença também é relatada em um estudo internacional^(12)^.

Sobre o ponto de corte do declínio da eTFG ≥ 25% em relação aos níveis basais em qualquer ponto de tempo realizado, em nossa amostra identificamos uma pequena variação de declínio entre as pessoas usuárias da PrEP nas 48 semanas de acompanhamento. Semelhante incidência é referida em um estudo realizado na França e no Canadá, no qual em seis meses de uso da PrEP apresentou-se um declínio ≥20% em 4% das pessoas em uso da PrEP^(17)^. Enquanto que em um estudo brasileiro, 1,8% das pessoas usuárias tiveram redução da eTFG > 30% em relação a linha de base^(11)^. Além disso, ressalta-se que aqueles que iniciam o uso da PrEP com eTFG <90mL/min/1,73 m^2^ tem um risco menor de haver um declínio > 25%^(28)^.

Em nosso estudo, identificamos que o declínio da eTFG > 25% em relação à linha basal das pessoas em uso da PrEP oral diária não foi significativamente associado à tubulopatia ao longo de 48 semanas, nem os marcadores de dano renal previram um declínio clinicamente relevante da eTFG. No estudo de revisão sistemática sobre a segurança da PrEP baseada em TDF, identificou-se que, mesmo que não havendo diferenças significativas no declínio da eTFG, os marcadores de disfunção tubular proximal individualmente pareciam estar associados a maiores chances de declínio ≥ 25% da eTFG^(26)^. Inclusive sugere que o monitoramento rotineiro desses marcadores de tubulopatia proximal não é uma abordagem eficiente, visto que sua presença é um evento raro. Entretanto, mostra-se prudente realizar avaliações mais frequentes em pessoas que evidenciarem tubulopatia proximal baseado na presença dos marcadores de dano renal^(26)^.

Diante disso, o recente Protocolo de Diretrizes Terapêuticas (PCDT) traz atualizações relevantes, onde as pessoas jovens e sem comorbidades serão avaliadas anualmente, enquanto as que tiverem 50 anos ou mais e aquelas que apresentarem fatores de risco de adoecimento renal como DM, HAS ou histórico de doença renal e/ou ter eTFG basal < 90 mL/min serão monitoradas semestralmente e as que não apresentarem nenhum destes perfis terão a avaliação anual da função renal^(9)^. Tal modificação da frequência de monitoramento renal pode ter um impacto positivo sobre a gestão da saúde pública, com a redução de custos com excessos de exames laboratoriais, além de promover uma maior adesão das pessoas usuárias.

As baixas incidências e a não associação dos fatores que se relacionam com a disfunção renal encontrada em nosso estudo não inviabilizam a relevância de avaliar a função renal, visto que as taxas de declínio podem ser leves, porém, as mesmas existem e são comprovadas pela nefrotoxicidade do TDF. Desse modo, faz-se necessário atentar-se ao monitoramento da função renal de forma regular e periódica conforme a faixa etária e a presença de fatores de risco para DRC existentes^(9,28)^. Reafirma-se, em conformidade com os estudos recentes, que diante da baixa incidência de declínio eTFG ≥ 25% em relação à eTFG basal encontrada, é válido considerar uma maior periodicidade do monitoramento da função renal, desde que as pessoas usuárias da PrEP sejam reagrupadas com cautela quanto o seu histórico pessoal de fatores de risco para DRC e faixa etária, assim como sejam constantemente reavaliadas quanto às possíveis mudanças que poderão existir na sua saúde durante o uso da PrEP^(10,29)^.

Vale frisar que, na literatura, ainda há uma escassez de estudos que avaliam o declínio da eTFG a longo prazo em pessoas usuárias da PrEP, visto que os estudos mais longos estão relacionados a um período de até três anos de acompanhamento^(4)^. Este é um possível foco relevante para pesquisas futuras, pois a ação nefrotóxica do TDF e o fato de agir no declínio progressivo da eTFG em pessoas vivendo com HIV já é comprovada cientificamente^(30)^.

Como limitação do estudo, destaca-se a ausência de informações obtidas nos prontuários em relação aos dados referentes aos antecedentes familiares, pessoais e uso de medicamentos, uma vez que tais dados são relevantes e poderiam estar relacionados ao risco de declínio da eTFG. Ademais, também houve dados ignorados de registro dos resultados dos exames laboratoriais que devem ser registrados no sistema eletrônico *Hygiaweb*. Apesar disto, os dados obtidos são os mesmos utilizados para o monitoramento das pessoas em uso da PrEP na prática clínica e permitiram realizar estudo de acompanhamento de 48 semanas, assim como encontrado em outros estudos de avaliação da função renal em pessoas em uso da PrEP.

Dentre as estratégias de ampliar o acesso ao uso da PrEP, nos estados e municípios brasileiros, e alcançar a meta da eliminação da infecção pelo HIV como problema de saúde pública no Brasil, o Ministério da Saúde incluiu o enfermeiro como integrante da equipe de saúde para prescrever a PrEP e realizar o seguimento clínico das pessoas usuárias da PrEP, no âmbito do SUS, conforme Protocolo Clínico e Diretrizes Terapêuticas da Profilaxia Pré-Exposição ao HIV (PCDT/PrEP) desde que estejam estabelecidos em programas de saúde pública e em rotina aprovada pela instituição de saúde^(9)^, com o objetivo de diminuir o número de novas infecções em consonância com os ODS^(9)^.

Cabe ressaltar que o reconhecimento da competência técnico-científica do enfermeiro expressa a relevância deste profissional para o avanço das políticas de prevenção e cuidado no contexto da infecção pelo HIV e configura uma ampliação do escopo da prática clínica de Enfermagem^(31)^. O enfermeiro deve incluir em todas as consultas de enfermagem, junto às pessoas usuárias da PrEP, a avaliação dos marcadores da função renal e a implementação de intervenções de enfermagem que envolvam o letramento em saúde sobre a importância do seguimento clínico e laboratorial, esclarecendo a relevância da avaliação função renal sobretudo entre as pessoas com maior risco de alteração^(31-32)^, visto que as características sociodemográficas e clínicas das pessoas são importantes para a implementação de estratégias personalizadas e adequadas de letramento em saúde^(33)^.

Destaca-se a importância da avaliação clínica de enfermagem no contexto da prescrição da PrEP e o acompanhamento integral às pessoas em uso de PrEP, incluindo a solicitação de exames para o monitoramento adequado da função renal. No monitoramento da função renal, o conhecimento clínico do enfermeiro é essencial para a identificação precoce de fatores de risco e a condução criteriosa dos processos de inclusão, manutenção e eventual descontinuação do uso da PrEP, assegurando a indicação desta estratégia de prevenção de forma apropriada e segura considerando a singularidade clínica de cada pessoa^(34)^.

O monitoramento da função renal das pessoas em uso da PrEP, proposto por este estudo, tem uma contribuição relevante para o avanço do conhecimento científico, visto que ainda há poucos estudos que foquem nos efeitos da PrEP nos marcadores da função renal ao longo do tempo, principalmente em âmbito nacional, contribuindo com os consensos sobre o uso desta importante estratégia de prevenção. Tal conhecimento reforça as recomendações do protocolo brasileiro, e apontam para a importância de intervenções de saúde e enfermagem com foco no monitoramento e autocuidado, particularmente entre as pessoas em uso da PrEP que possuem fatores de risco para disfunção renal.

## Conclusão

As pessoas em uso da PrEP que foram acompanhadas por 48 semanas em sua maioria eram homens, cis, homossexuais, brancos, com alta escolaridade e jovens com idade média de ± 34 anos. A presença de marcadores de dano renal prediz uma baixa lesão renal em pessoas usuárias da PrEP. Dentre os marcadores a proteinúria teve um aumento até a 12ª semana (2,6%), mantendo uma pequena variação até a 48ª semana, todos os casos foram eventos leves ou grau 1 de proteinúria. A glicosúria foi ainda mais rara, entretanto, um participante teve evolução de grau 2 para grau 4.

Em nosso estudo, não identificamos alterações na média de creatinina sérica na avaliação das semanas de acompanhamento, entretanto a eTFG calculada por Cockcroft-Gault apresentou declínio leve e progressivo na 12ª, 36ª e 48ª semanas, enquanto o CKD-EPI reduziu apenas nas semanas 36 e 48. A eTFG calculada por CG em comparação com CKD-EPI permaneceu com uma diferença em torno de 20 mL/min em todo período do estudo, sendo a eTFG calculada por CG mais alta que por CKD-EPI.

Além disso, os resultados desta pesquisa ratificam a baixa incidência de eventos que caracterizam disfunção renal. Durante as 48 semanas de acompanhamento do estudo, entre 3% e 4,4% da população apresentou declínio da eTFG igual ou superior a 25%, além de apresentar eventos leves e isolados de proteinúria, glicosúria, uricosúria e cilindrúria em pessoas usuárias da PrEP. Não houve associação significativa entre o declínio da eTFG ≥ 25% e presença de um dos marcadores de dano renal estudados.

## Data Availability

Os conjuntos de dados relacionados a este artigo estarão disponíveis mediante solicitação ao autor correspondente.

# Translation: Efecto de los antirretrovirales en la función renal de las personas en uso de la profilaxis previa a la exposición al VIH*

**DOI:** 10.1590/1518-8345.7280.4733

**Authors:** Priscila Silva Pontes-Pereira, Rodrigo de Carvalho Santana, Elucir Gir, Renata Karina Reis

**Affiliations:** 1 Universidade de São Paulo, Escola de Enfermagem de Ribeirão Preto, Centro Colaborador de la OPS/OMS para el Desarrollo de la Investigación en Enfermería, Ribeirão Preto, SP, Brasil.; 2 Universidade de São Paulo, Faculdade de Medicina de Ribeirão Preto, Ribeirão Preto, SP, Brasil.

**Keywords:** Profilaxis Pre-Exposición, Antirretrovirales, Tenofovir, Enfermedades Renales, Monitoreo Fisiológico, Tasa de Filtración Glomerular

## Abstract

evaluar el efecto de los antirretrovirales en la función renal de las personas en uso de la profilaxis previa a la exposición al VIH.

delineamiento descriptivo tipo encuesta longitudinal, realizado en la cohorte retrospectiva por 48 semanas. La muestra fue de 203 participantes. El muestreo fue no probabilístico por conveniencia. Se analizaron variables sociodemográficas, clínicas y de laboratorio. Se utilizó frecuencia absoluta, relativa, media, desviación estándar, mínima y máxima, prueba exacta de Fisher, prueba de Friedman, nivel de significancia <5%.

predominantemente hombres cis (86,2%), color de piel blanca (69,1%), alta escolaridad (76,7%) y edad media de ± 34 años. La presencia de marcadores de daño renal fue baja. La proteinuria tuvo un aumento hasta la semana 12 (2,6%), manteniendo una pequeña variación hasta la semana 48. La disminución de la estimación de la Tasa de Filtración Glomerular (eTFG) ≥25% en relación con los niveles basales fue de 4,4%, 3%, 4,4% y 3% en las semanas 12, 24, 36 y 48 respectivamente. No hubo asociación significativa entre la disminución de la eTFG ≥25% y la presencia de uno de los marcadores de daño renal.

se ratifica la baja incidencia de eventos que caracterizan disfunción renal, como la disminución de la eTFG ≥25% y la presencia de eventos leves y aislados de proteinuria, glucosuria, uricosuria y cilindruria en 48 semanas de seguimiento.

## Destacados

**(1)** Pocos estudios sobre disfunción renal en usuarios de la profilaxis previa a la exposición al VIH. **(2)** Baja incidencia de disfunción renal en usuarios de la PrEP durante 48 semanas. **(3)** Baja incidencia de marcadores de daño renal durante 48 semanas de seguimiento. **(4)** El conocimiento clínico del enfermero para el monitoreo de la función renal es esencial.

## Introducción

El debilitamiento del progreso en la respuesta al Virus de la Inmunodeficiencia Humana (VIH) en los últimos años refleja una preocupante reemergencia de la epidemia de la infección por VIH^(1)^. Los informes globales señalan un total de 1,3 millones de nuevas infecciones por VIH en 2023^(2)^ y, si dichas tendencias se mantienen, 1,2 millones de personas serán infectadas por el virus en 2025, es decir, tres veces más que la meta para este año^(3)^.

De acuerdo con las metas propuestas por las Naciones Unidas para el logro de los Objetivos de Desarrollo Sostenible (ODS), se busca reducir la incidencia de nuevos casos de VIH en el mundo a un total de 200.000 y eliminar la epidemia del sida como problema de salud pública para 2030^(2)^. En este contexto, se observa un cambio en el paradigma de la prevención de la transmisión sexual del VIH derivado de los avances obtenidos en los últimos años con la propuesta de la prevención combinada, la cual incluye la recomendación y disponibilidad de diversas estrategias, con énfasis en las intervenciones biomédicas basadas en el uso de medicamentos antirretrovirales, como la Profilaxis Previa a la Exposición al VIH (PrEP), considerada uno de los avances más importantes recientes en la prevención de la infección por VIH^(4)^. La PrEP se basa en la combinación de antirretrovirales, siendo una de las más comunes la asociación de Tenofovir Disoproxil Fumarato (TDF) y Emtricitabina (FTC) por vía oral (TDF/FTC) en dosis fija de 300/200 mg^(4)^.

A pesar de los diversos beneficios del uso regular de la PrEP, las personas que la utilizan deben ser monitoreadas en cuanto a los posibles efectos adversos de su uso continuo^(5)^. Cabe resaltar que ya está establecido en la literatura que el TDF está asociado a lesión tubular renal proximal, obstrucción inducida por cristales o nefritis intersticial^(6)^, necrosis tubular aguda tóxica con dismorfia y ensanchamiento mitocondrial distintivo^(7)^. Ensayos clínicos han demostrado que dicho fármaco se relaciona con un mayor riesgo de Insuficiencia Renal Aguda (IRA), proteinuria nefrogénica, Diabetes Insípida, síndrome de Fanconi y Enfermedad Renal Crónica (ERC)^(8-9)^.

De esta manera, la preocupación por la disfunción renal entre personas que hacen uso de la PrEP es relevante para la práctica clínica. Diferentes estudios describen una disminución de la estimación de la Tasa de Filtración Glomerular (eTFG) por el TDF en personas usuarias de PrEP, variando del 1% al 15%^(10-14)^. Esta amplia variabilidad puede estar relacionada con las diferentes características poblacionales investigadas, incluyendo sexo, etnia y edad^(10-14)^. Otros indicadores de lesión renal son reportados, como la disminución de la eTFG ≥25% en relación con la tasa basal^(15)^; presencia de marcadores de tubulopatía proximal como creatininuria, proteinuria no albúmina, glucosuria euglucémica, aumento de la excreción urinaria de fosfato, aumento de la excreción urinaria de ácido úrico^(15-17)^.

La evaluación cuidadosa de los efectos adversos a nivel renal es esencial para maximizar los beneficios y minimizar los riesgos^(9)^. El seguimiento rutinario de la función renal es crítico en personas que reciben TDF, dado que el uso continuo de un fármaco antirretroviral nefrotóxico será incluido en la vida cotidiana de estas personas como un método profiláctico para la prevención del VIH^(9)^.

Ante la temática expuesta y la comprobada relevancia internacional de la cuestión relacionada con la acción de un antirretroviral nefrotóxico utilizado como agente profiláctico de prevención del VIH, desde la indicación del método preventivo hasta la frecuencia de evaluación, así como sus impactos en la adherencia medicamentosa, clínicos y económicos, se justifica el actual interés entre científicos y enfermeros clínicos para alcanzar consensos sobre el uso de la PrEP a nivel renal. Por lo tanto, el objetivo del estudio presentado fue evaluar el efecto de los antirretrovirales en la función renal de las personas en uso de la PrEP.

## Método

### Delineamento del estudio

Se trata de un estudio de cohorte retrospectiva realizado en personas seronegativas al VIH en uso de la PrEP durante 48 semanas. El período de 48 semanas de seguimiento de las personas en uso de la PrEP correspondió a quienes iniciaron su uso entre enero de 2018 y enero de 2021. La recolección de datos se llevó a cabo en un período de cuatro meses, comprendido entre mayo y agosto de 2022.

### Lugar de recolección de datos

Los participantes elegibles fueron identificados entre las personas usuarias de cuatro Servicios de Atención Especializada (SAE) correspondientes a las regiones central, este, sur y norte, ubicados en el sudeste de Brasil, específicamente en un municipio del interior del estado de São Paulo.

### Población

Inicialmente fueron identificadas, en los SAE, 614 personas que iniciaron el uso de la PrEP entre enero de 2018 y enero de 2021, distribuidas en los SAE de la región central (419), este (52), sur (61) y norte (64).

### Criterio de selección

Entre los criterios de selección de este estudio, se consideraron los siguientes de inclusión: personas en uso de la PrEP con edad ≥18 años; disponibilidad de registros en la historia clínica de, al menos, una consulta de retorno y cuatro de seguimiento trimestrales, así como de los resultados de los exámenes de laboratorio; inicio de la PrEP entre enero de 2018 y enero de 2021; función renal normal (creatinina sérica ≤1,3 mg/dL para hombres / ≤1,1 mg/dL para mujeres y eTFG ≥60 mL/min) antes de iniciar la PrEP. Los criterios de exclusión fueron: no haber completado el período de seguimiento regular de 48 semanas; ausencia de registro completo de la información y de los resultados de laboratorio en la historia clínica de los participantes; y tener diagnóstico de diabetes mellitus (DM).

### Definición de la muestra

La muestra fue no probabilística, consecutiva, compuesta por todas las personas usuarias de la PrEP que iniciaron su uso entre enero de 2018 y enero de 2021. El tamaño de la muestra incluyó la población elegible en el período de recolección de datos, es decir, se consultaron y evaluaron de forma manual y no probabilística todos los registros de las historias clínicas de las personas en uso de la PrEP en cuanto a los criterios de elegibilidad del estudio, resultando en un total de 203 participantes, pertenecientes a los SAE de las regiones central (158), este (20), sur (14) y norte (11). Cabe resaltar la existencia de datos faltantes en las historias clínicas de los participantes a lo largo de las 48 semanas de seguimiento.

### Variables del estudio

Las variables consideradas para este estudio fueron sociodemográficas, clínicas generales y de laboratorio.

Las variables sociodemográficas comprendían: sexo de nacimiento (femenino, masculino), orientación sexual (heterosexual, homosexual, bisexual, ignorado); identidad de género (hombre cisgénero, hombre transgénero, mujer cisgénero, mujer transgénero, travesti, pansexual, otros); edad en años cumplidos; grupo etario (20-39 años / 40-49 años / ≥50 años); color de piel autodeclarado (blanco, negro, amarillo, pardo, no declara); escolaridad (ninguno / sin educación formal / 1 a 3 años de estudio / 4 a 7 años de estudio / 8 a 11 años de estudio / ≥12 años de estudio); situación de calle (sí, no).

En relación con las variables clínicas generales y de laboratorio se consideraron para el estudio: antecedentes de hipertensión arterial sistémica (HAS) (sí, no); dislipidemia (sí, no); uso de medicamentos antihipertensivos (sí, no); dosificación de creatinina sérica (mg/dL); eTFG; examen de orina para evaluación de proteinuria [ausente, presente-trazas, una cruz (1+), dos cruces (2+), tres cruces o más (3+)], glucosuria [ausente, presente (1+), dos cruces (2+), tres cruces o más (3+)], ácido úrico (presente, ausente), cilindros hialinos (presente, ausente) y cilindros granulosos (presente, ausente).

### Instrumentos utilizados para la recolección de la información

Para la recolección de datos se utilizó un cuestionario semiestructurado sometido a validación teórica de apariencia y contenido por cinco expertos en el área, quienes lo evaluaron en cuanto a la pertinencia, facilidad de comprensión y relevancia de las variables seleccionadas.

Tanto el cuestionario de recolección de datos como el cuestionario de validación fueron enviados por correo electrónico a cinco jueces especialistas en infectología, nefrología y metodología, identificados a través de currículum Lattes, con un plazo establecido de 15 días. Tras el análisis y los ajustes necesarios, se realizó un estudio piloto con 15 historias clínicas de personas en uso de la PrEP en uno de los SAE. Dicho instrumento se utilizó específicamente con el fin de alcanzar los objetivos de este estudio. Cabe destacar que las 15 historias clínicas del ensayo piloto no fueron incluidas en la muestra final del estudio.

Para la evaluación de la función renal se utilizó la eTFG calculada mediante ecuaciones matemáticas empíricas basadas en la dosificación de creatinina sérica: *Chronic Kidney Disease Epidemiology Collaboration* (CKD-EPI) (mL/min/1,73 m²)^(18)^ y Cockcroft-Gault (CG) (mL/min)^(19)^, empleando como herramienta la calculadora de la aplicación de la *National Kidney Foundation* (NKF)^(20)^.

Para evaluar la escala de gravedad de los eventos adversos, específicamente los marcadores de daño renal (proteinuria, glucosuria y cilindruria), se utilizó la escala de gravedad de eventos adversos en adultos empleada por la *France Recherche Nord et Sud Sida-HIV et Hepatites* [Agencia Nacional de Investigación en Sida y Hepatitis (ANRS)], que clasifica los eventos en: Grado 1 o evento leve (trazas a 1+); Grado 2 o evento moderado (2+) y Grado 3 o evento severo (3+ o más)^(21)^.

Para identificar la disminución de la función renal se utilizó el acrónimo RIFLE: *risk* (R: riesgo), *injury* (I: lesión), *failure* (F: fallo), *loss* (L: pérdida mantenida de la función) y *end-stage kidney disease* (E: insuficiencia renal terminal). El RIFLE establece que las tres primeras clases más sensibles y relacionadas con los grados de gravedad de la disfunción renal se evalúan mediante cambios relativos en el valor del nivel sérico de creatinina o en la TFG a partir de un valor basal^(22)^. Como marcador de lesión renal clínicamente significativa, se consideró como punto de corte una disminución significativa de la eTFG ≥25% en relación con los niveles basales en cualquier momento^(22)^.

### Recolección de datos

Los datos fueron recolectados por la investigadora y una asistente de investigación tras capacitación teórica y práctica. La recolección de datos se dividió en cinco momentos: línea de base (antes de iniciar la PrEP) y cuatro seguimientos clínicos y de laboratorio trimestrales, totalizando un período de seguimiento de 48 semanas.

Se obtuvieron datos sociodemográficos, clínicos generales y de exámenes de laboratorio, así como el cálculo de la eTFG mediante las ecuaciones CKD-EPI y CG.

Los datos fueron recolectados a través de: formulario de registro de usuario de la PrEP, ficha de primera atención, ficha de retorno a 30 días, ficha de seguimiento clínico en PrEP y los exámenes de laboratorio trimestrales registrados en el sistema *Hygiaweb*, un *software* de gestión en salud utilizado en el municipio donde se encuentran los SAE, desarrollado para automatizar procesos y promover la integración entre las unidades de salud de la red pública, cuyas funciones incluyen la disponibilidad en línea de los resultados de los exámenes de laboratorio realizados en los servicios de salud.

Para obtener la eTFG mediante las ecuaciones CKD-EPI y CG, fue necesaria la información sobre sexo, edad y creatinina sérica; y para CG, sexo, edad, peso y creatinina sérica respectivamente. Para la identificación de los marcadores de daño renal se evaluaron los resultados de una muestra única de orina recolectada por la mañana, la cual fue analizada trimestralmente, incluyendo: parámetros químicos como ácido úrico y biomarcadores urinarios como proteinuria y glucosuria (cruces) y cilindruria – cilindros hialinos y granulosos (presente o ausente). En nuestro estudio, la proteinuria identificada en exámenes de laboratorio fue cualitativa, clasificada en cruces (trazas, 1+, 2+, 3+ y 4+).

### Tratamiento y análisis de los datos:

Los datos del estudio fueron recolectados y gestionados utilizando la herramienta de captura electrónica de datos *Research Electronic Data Capture* (REDCap), alojada y disponible por la institución proponente^(23)^. El REDCap es una plataforma de *software* segura y basada en la *web*, diseñada para apoyar la captura de datos para estudios de investigación, proporcionando una interfaz intuitiva para la captura de datos validados y pistas de auditoría para rastrear la manipulación de datos y procedimientos^(23)^.

Los datos fueron procesados y analizados mediante el *software Statistical Package for the Social Sciences* (SPSS), versión 26.0. Se utilizaron estadísticas descriptivas por medio de frecuencia absoluta (n) y relativa (%), media, desviación estándar (DE), mínima y máxima, y la prueba de asociación exacta de Fisher. Para las variables significativas se empleó la regresión logística para calcular la razón de probabilidades, *Odds Ratio* (OR). La prueba exacta de Fisher también se adoptó para medir la asociación entre la reducción de la función renal y los marcadores de daño renal. Se aplicó la prueba de normalidad de Kolmogorov-Smirnov para medir la simetría entre las tasas del CKD-EPI (mL/min/1,73 m²) y Cockcroft-Gault (mL/min). La prueba de Friedman se utilizó para el análisis multivariado entre los momentos analizados. Para todos los análisis, se consideró un nivel de significancia <5%.

### Aspectos éticos

El estudio fue conducido de acuerdo con las normas legales y éticas recomendadas por la Resolución 466/2012, siendo aprobado por el Comité de Ética en Investigación de la institución responsable bajo el protocolo CAEE 31543020.3.0000.5393, en fecha 08/07/2020.

## Resultados

Los resultados presentados corresponden a 203 personas con alto riesgo de adquirir el VIH en uso de la PrEP diaria, acompañadas durante 12 meses. En relación con las variables sociodemográficas mostradas en la Tabla 1, se verificó que 158 (77,8%) de los participantes provenían del SAE Central, siendo el grupo etario más frecuente el de 20 a 39 años, con una media de edad de 34,7±7,7 años. La mayoría pertenecía al sexo de nacimiento masculino 185 (91,1%), de los cuales 175 (86,2%) eran hombres cisgénero. En cuanto a la orientación sexual, predominaban los participantes homosexuales, totalizando 161 (79,3%) personas.

En relación con el color de piel, 134 (66,0%) se autodeclaraban blancos, seguidos por pardos, 38 (18,7%). Solo un (0,5%) participante se encontraba en situación de calle. En cuanto al nivel educativo, 133 (65,5%) tenían más de 12 años de estudio, seguidos por 8 a 11 años de estudio, 31 (15,3%) (Tabla 1).

**Tabla 1-:** Perfil sociodemográfico y clínico de personas con alto riesgo de adquirir el VIH en uso de la profilaxis previa a la exposición (n = 203). Ribeirão Preto, SP, Brasil, 2022

**Variables**	**n (%)**
**Distrito de salud**	
Central	158 (77,8)
Este	20 (9,9)
Sur	14 (6,9)
Norte	11 (5,4)
**Grupo etario**	
20-39 años	158 (77,8)
40-49 años	37 (18,3)
≥50 años	8 (3,9)
**Sexo de nascimiento**	
Femenino	18 (8,9)
Masculino	185 (91,1)
**Identidad de género**	
Mujer cis	18 (8,9)
Hombre cis	175 (86,2)
Mujer trans	6 (2,9)
Travesti	4 (2,0)
**Orientación sexual**	
Heterosexual	25 (12,3)
Homosexual	161 (79,3)
Bisexual	17 (8,4)
**Color de piel**	
Ignorado	10 (4,9)
Blanco	134 (66,0)
Negro	19 (9,4)
Pardo	38 (18,7)
Amarillo	2 (1,0)
**Situación de calle**	
Ignorada	4 (2,0)
No	198 (97,5)
Sí	1 (0,5)
**Nivel educativo**	
Ignorado/sin información	31 (15,3)
Ninguno/sin educación formal	0 (0,0)
1 a 3 años de estudio	0 (0,0)
4 a 7 años de estudio	8 (3,9)
8 a 11 años de estudio	31 (15,3)
Más de 12 años de estudio	133 (65,5)
**HAS** *	
No	199 (98,0)
Sí	4 (2,0)
**Uso de antihipertensivo**	
No	199 (98,0)
Sí	4 (2,0)
**Dislipidemia**	
No	201 (99,0)
Sí	2 (1,0)
**Total**	203 (100)

*HAS = Hipertensión Arterial Sistémica

En cuanto a la evaluación de la función renal, se observó una baja incidencia de marcadores de lesión renal en el examen de orina de los participantes durante las 48 semanas de seguimiento. En ningún participante se detectó proteinuria moderada o grave durante todo el seguimiento. La presencia de proteinuria leve (trazas o 1+) se observó en un bajo porcentaje de participantes y se mantuvo relativamente estable a lo largo de las 48 semanas. Se observó un discreto aumento en la semana 36 con retorno a los valores basales al final de las 48 semanas (Tabla 2).

**Tabla 2-:** Monitoreo de los marcadores de lesión renal de acuerdo con el tiempo de uso de la profilaxis previa a la exposición al VIH (n = 203). Ribeirão Preto, SP, Brasil, 2022

**Variables**	**Basal n (%)**	**12 semanas n (%)**	**24 semanas n (%)**	**36 semanas n (%)**	**48 semanas n (%)**
**Proteinuria**					
Ignorado	22 (10,8)	9 (4,4)	13 (6,4)	12 (5,9)	14 (6,8)
Ausente	163 (80,3)	174 (85,7)	177 (87,2)	168 (82,7)	178 (87,7)
Trazas*	16 (7,9)	15 (7,4)	10 (4,9)	19 (9,4)	8 (4,0)
1+*	2 (1,0)	5 (2,5)	3 (1,5)	4 (2,0)	3 (1,5)
2+ ^†^	0	0	0	0	0
3+ ^‡^	0	0	0	0	0
4+ ^‡^	0	0	0	0	0
**Glicosuria**					
Ignorado	17 (8,4)	9 (4,4)	8 (4,0)	8 (4,0)	10 (4,9)
Ausente	185 (91,1)	192 (94,6)	194 (95,5)	194 (95,5)	190 (93,6)
Trazas o 1 +*	0	1 (0,5)	0	0	2 (1,0)
2 + ^†^	1 (0,5)	0	0	0	1 (0,5)
3 + ^‡^	0	0	0	0	0
4 + ^‡^	0	1 (0,5)	1 (0,5)	1 (0,5)	0
Ácido úrico					
Ignorado	18 (8,9)	10 (4,9)	10 (4,9)	11 (5,4)	10 (4,9)
Ausente	185 (91,1)	191 (94,1)	193 (95,1)	192 (94,6)	193 (95,1)
Presente	0	2(1,0)	0	0	0
**Cilindro hialino**					
Ignorado	18 (8,9)	10 (4,9)	10 (4,9)	11 (5,4)	10 (4,9)
Ausente	185 (91,1)	192 (94,6)	191 (94,1)	192 (94,6)	192 (94,6)
Presente	0	1 (0,5)	2 (1,0)	0	1 (0,5)
**Cilindro granuloso**					
Ignorado	18 (8,9)	10 (4,9)	10 (4,9)	11 (5,4)	10 (4,9)
Ausente	185 (91,1)	193 (95,1)	193 (95,1)	192 (94,6)	192 (94,6)
Presente	0	0	0	0	1 (0,5)

Proteinuria y Glicosuria: *Trazas o 1+ = Grado 1 (leve); ^†^2+= Grado 2 (moderado); ^‡^3+o 4+=Grado 3 (grave)

En cuanto a la evaluación trimestral de la función renal mediante la eTFG (Cockcroft-Gault y CKD-EPI), no se observó una reducción significativa, manteniéndose normal a lo largo del tiempo de exposición al TDF. Cabe resaltar que la eTFG por Cockcroft-Gault y CKD-EPI difirieron considerablemente entre sí desde el momento basal, cuando se observó una media de 121,42±33,46 y 100,42±17,71, respectivamente. Esta diferencia perduró hasta las 48 semanas de seguimiento, con una media de 121,42±33,46 y 101,63±16,69, respectivamente. La creatinina sérica media se mantuvo normal durante el período de seguimiento (Tabla 3).

**Tabla 3-:** Comparación de la creatinina sérica, *clearance* de creatinina, eTFG (Cockcroft-Gault)* y eTFG (CKD-EPI)^†^ de acuerdo con el tiempo de uso de la profilaxis previa a la exposición al VIH (n = 203). Ribeirão Preto, SP, Brasil, 2022

**Variables**	**Media±DE** ^‡^	**IC (95%)**	**Min-Máx**	**P-valor** ^§^
**Creatinina Sérica**				
Basal	1,01±0,19	(0,98-1,04)	(0,50-1,70)	0,244
12 semanas	1,00±0,18	(0,98-1,03)	(0,52-1,80)	
24 semanas	0,98±0,17	0,96-1,01	(0,50-1,50)	
36 semanas	0,99±0,19	0,96-1,01	(0,50-1,60)	
48 semanas	1,00±0,17	0,97-1,02	(0,60-1,53)	
eTFG (CG)*				
Basal	121,42 ± 33,46	116,79-126,05	(64-275)	0,440
12 semanas	120,32 ± 31,19	116,01-124,64	(52-238)	
24 semanas	123,36 ± 32,62	118,85-127,88	(61-265)	
36 semanas	123,17 ± 31,80	118,77-127,57	(62-242)	
48 semanas	121,43 ± 31,44	117,08-125,78	(62-277)	
**eTFG (CKD-EPI)** ^†^				
Basal	100,42±17,71	97,97-102,88	(54-147)	0,510
12 semanas	100,67±16,42	98,39-102,94	(50-148)	
24 semanas	102,59±16,27	100,34-104,84	(64-145)	
36 semanas	102,32±17,70	99,87-104,77	(58-132)	
48 semanas	101,63±16,69	99,32-103,94	(60-130)	

*eTFG = Estimación de la Tasa de Filtración Glomerular por Cockcroft-Gault (mL/min); ^†^eTFG = Estimación de la Tasa de Filtración Glomerular por CKD-EPI (mL/min/1,73m²); ^‡^DE = Desviación Estándar; ^§^P-valor = Prueba de Friedman, con un nivel de significancia del 5%

La distribución de los participantes que presentaron disminución de la eTFG en los cuatro momentos de evaluación (semanas 12, 24, 36 y 48) se muestra en la Figura 1, la cual indica que la proporción de participantes con disminución de la eTFG ≥25% en las semanas 12 y 36 de uso de la PrEP fue del 4,4%. También se observó una oscilación con mejora en la tasa de reducción en las semanas 24 y 48, con un 3%.

**Figura 1-:** Distribución de las reducciones de la estimación de la tasa de filtración glomerular en relación con la evaluación basal en las semanas 12, 24, 36 y 48 de seguimiento (n = 203). Ribeirão Preto, SP, Brasil, 2022

Nota: El n de participantes entre las semanas de seguimiento corresponde a aquellos en los que hubo disminución de la función renal

La Tabla 4 muestra la distribución de los participantes que presentaron disminución de la eTFG ≥25% y <25% y que tuvieron presencia de marcadores de lesión renal; por lo tanto, no todos los participantes presentaron disminución de la eTFG ni presencia de marcadores de lesión renal. De los nueve participantes con disminución de la eTFG en las primeras 12 semanas de uso de la PrEP, el 25% (n=2) presentaban proteinuria y disminución de la eTFG ≥25%. Se observa también que la mayoría de las personas usuarias de la PrEP con proteinuria presente tuvieron una disminución de la eTFG <25%.

**Tabla 4-:** Asociación entre la disminución de la estimación de la Tasa de Filtración Glomerular (eTFG) y los marcadores de lesión renal de acuerdo con el tiempo de uso de la profilaxis previa a la exposición al VIH. Ribeirão Preto, SP, Brasil, 2022

**Marcadores de lesión renal**	**Disminución de la estimación de la Tasa de Filtración Glomerular (eTFG)**											
	**12 semanas**		p*	**24 semanas**		p*	**36 semanas**		p*	**48 semanas**		p*
	≥25% n (%)	<25% n (%)		≥25% n (%)	<25% n (%)		≥25% n (%)	<25% n (%)		≥25% n (%)	<25% n (%)	
**Proteinuria**												
Ausente	6 (75,0)	90 (92,8)	0,084	5 (100)	172 (93,0)	0,539	8 (88,9)	86 (88,7)	0,983	4 (80)	97 (94,2)	0,209
Presente ^†^	2 (25,0)	7 (7,2)		0	13 (7,0)		1 (11,1)	11 (11,3)		1 (20)	6 (5,8)	
**Glicosuria**												
Ausente	8 (100)	100 (100)	-	5 (100)	189 (99,5)	0,871	9 (100)	98 (100)	-	5 (100)	102 (99,0)	0,825
Presente ^‡^	0	0		0	1 (0,5)		0	0		0	1 (1,0)	
Ácido úrico												
Ausente	8 (100)	99 (100)	-	5 (100)	188 (100)	-	9 (100)	97 (100)	-	5 (100)	103 (100)	-
Presente	0	0		0	0		0	0		0	0	
**Cilindro hialino**												
Ausente	8 (100)	99 (100)	-	5 (100)	186 (98,9)	0,817	9 (100)	97 (100)	-	5 (100)	102 (99,0)	0,049
Presente	0	0		0	2 (1,1)		0	0		0	1 (1)	
**Cilindro granuloso**												
Ausente	8 (100)	99 (100)	-	5 (100)	188 (100)	-	9 (100)	97 (100)	-	5 (100)	102 (99,0)	0,825
Presente	0	0		0	0		0	0		0	1 (1)	

*p-valor para la prueba de Friedman; ^†^Presente y ^‡^Presente: sumatoria de todos los resultados presentes (trazas, una cruz, dos cruces, tres cruces y cuatro cruces). El n presentado en cada marcador de lesión renal durante las 48 semanas de seguimiento es menor que el número total de participantes, n=203

## Discusión

En este estudio fue posible identificar un predominio de personas usuarias de la PrEP de sexo masculino, cis, homosexuales, de piel blanca, con alto nivel educativo y jóvenes. La presencia de marcadores de daño renal capaces de predecir la evolución de lesión renal fue baja en un período de 48 semanas de seguimiento. Además, los resultados de esta investigación ratifican la baja incidencia de eventos que caracterizan disfunción renal, con presencia de eventos leves y aislados de proteinuria, glucosuria, uricosuria y cilindruria en personas usuarias de PrEP. No hubo asociación significativa entre la disminución de la eTFG ≥25% y la presencia de alguno de los marcadores de daño renal estudiados.

A partir de la caracterización sociodemográfica de este estudio fue posible identificar un predominio de personas usuarias de la PrEP de sexo de nacimiento masculino y grupo etario joven, con una media de 34 años. Estos perfiles también fueron evidenciados en otros estudios que evaluaron la función renal de personas en uso de la PrEP a nivel internacional^(13,19)^. Asimismo, la disminución de la función renal entre las personas de mayor edad que iniciaron el uso de TDF/FTC fue observada en una cohorte retrospectiva^(24)^.

En relación con el monitoreo de los marcadores de daño renal en 48 semanas, identificamos que la proporción de proteinuria y glucosuria fue baja. Este hallazgo también fue identificado en un estudio realizado con 1.589 hombres que tienen sexo con hombres, en el que el 12% presentó proteinuria y el 1% glucosuria^(25)^. En relación con las primeras 12 semanas de uso de la PrEP, una cohorte prospectiva presentó una incidencia del 15% de proteinuria con respecto a la línea de base (p<0,0001)^(12)^, lo que difiere de nuestros hallazgos, en los cuales se presentó proteinuria en el 2,6% de la población en uso de la PrEP.

El estudio *Partners PrEP*, que realizó un análisis de sensibilidad, observó patrones similares en la distribución de glucosuria entre el grupo TDF/FTC y el grupo placebo; sin embargo, la excreción de proteínas y el aumento de la excreción de ácido úrico en la orina fueron predominantes en las personas en uso de la PrEP^(26)^. Aunque no hubo diferencia en el riesgo de tubulopatía proximal, definida como ≥2 indicadores de daño renal, los participantes del brazo TDF/FTC tuvieron un mayor riesgo de proteinuria tubular en comparación con placebo^(26)^. Otros investigadores también sugieren que la presencia de marcadores de daño renal después de seis meses de uso de la PrEP puede resultar en disfunción subclínica del túbulo^(27)^.

En relación con la eTFG, a pesar de que en nuestro estudio hubo una reducción de su media calculada por CG y CKD-EPI, la disminución no fue significativa, lo que coincide con el proyecto demostrativo multicéntrico PrEP Brasil, en el cual se identificó una reducción significativa en la eTFG de −3,46 ± 13,29 (p<0,001) en la semana 4 con respecto a la evaluación basal; sin embargo, no hubo diferencia estadística entre las variaciones en las semanas posteriores^(10)^. Este perfil también fue identificado en Australia, en participantes del estudio EPIC-NSW, donde se registró una caída inicial significativa (p<0,05) y un descenso gradual y progresivo, aunque no se reportó insuficiencia renal en un período de 18 meses de observación^(28)^.

También fue posible identificar que entre la eTFG calculada por CG y CKD-EPI hubo una amplia diferencia entre sus medias desde la línea de base hasta la semana 48, de 21; 19,65; 20,78; 20,85 y 19,8 mL/min/1,73m² en los momentos basal, semanas 12, 24, 36 y 48, respectivamente. Esta diferencia también es reportada en un estudio internacional^(12)^.

Con respecto al punto de corte de la disminución de la eTFG ≥25% en relación con los niveles basales en cualquier momento, en nuestra muestra identificamos una pequeña variación de disminución entre las personas usuarias de la PrEP durante las 48 semanas de seguimiento. Una incidencia similar fue referida en un estudio realizado en Francia y Canadá, en el cual, en seis meses de uso de la PrEP, se presentó una disminución ≥20% en el 4% de las personas en uso de la PrEP^(17)^. En un estudio brasileño, el 1,8% de las personas usuarias tuvo una reducción de la eTFG >30% con respecto a la línea de base^(11)^. Además, se resalta que quienes inician el uso de la PrEP con eTFG <90 mL/min/1,73m² tienen un menor riesgo de presentar una disminución >25%^(28)^.

En nuestro estudio identificamos que la disminución de la eTFG >25% en relación con la línea basal en personas en uso de la PrEP oral diaria no estuvo significativamente asociada a tubulopatía a lo largo de 48 semanas, ni los marcadores de daño renal predijeron una disminución clínicamente relevante de la eTFG. Un estudio de revisión sistemática sobre la seguridad de la PrEP basada en TDF identificó que, incluso sin diferencias significativas en la disminución de la eTFG, los marcadores de disfunción tubular proximal, de manera individual, parecían estar asociados a mayores probabilidades de disminución ≥25% de la eTFG^(26)^. Incluso sugiere que el monitoreo rutinario de estos marcadores de tubulopatía proximal no es un enfoque eficiente, dado que su presencia es un evento raro. Sin embargo, se considera prudente realizar evaluaciones más frecuentes en personas que presenten tubulopatía proximal, basada en la presencia de marcadores de daño renal^(26)^.

Ante esto, el reciente Protocolo de Directrices Terapéuticas (PCDT) presenta actualizaciones relevantes, en las que las personas jóvenes y sin comorbilidades serán evaluadas anualmente, mientras que quienes tengan 50 años o más, así como aquellos que presenten factores de riesgo de enfermedad renal como DM, HAS o antecedente de enfermedad renal y/o eTFG basal <90 mL/min, serán monitoreados semestralmente; quienes no presenten ninguno de estos perfiles tendrán la evaluación anual de la función renal^(9)^. Tal modificación en la frecuencia del monitoreo renal puede tener un impacto positivo en la gestión de la salud pública, con la reducción de costos derivados del exceso de exámenes de laboratorio, además de promover una mayor adherencia de las personas usuarias.

Las bajas incidencias y la no asociación de los factores relacionados con la disfunción renal encontradas en nuestro estudio no invalidan la relevancia de evaluar la función renal, dado que las tasas de disminución pueden ser leves, pero existen y están comprobadas por la nefrotoxicidad del TDF. De este modo, es necesario prestar atención al monitoreo de la función renal de forma regular y periódica, conforme al grupo etario y a la presencia de factores de riesgo para ERC existentes^(9,28)^. Se reafirma, al igual que en estudios recientes, que, dada la baja incidencia de disminución de la eTFG ≥25% en relación con la eTFG basal encontrada, es válido considerar una mayor periodicidad del monitoreo de la función renal, siempre que las personas usuarias de la PrEP sean reagrupadas con cautela según su historial personal de factores de riesgo para ERC y grupo etario, así como sean constantemente reevaluadas respecto a posibles cambios en su salud que puedan ocurrir durante el uso de la PrEP^(10,29)^.

Cabe destacar que en la literatura aún existe una escasez de estudios que evalúen la disminución de la eTFG a largo plazo en personas usuarias de la PrEP, ya que los estudios más prolongados abarcan un período de hasta tres años de seguimiento^(4)^. Este es un posible foco relevante para investigaciones futuras, dado que la acción nefrotóxica del TDF y su efecto sobre la disminución progresiva de la eTFG en personas que viven con VIH ya está científicamente comprobado^(30)^.

Como limitación del estudio, se resalta la ausencia de información obtenida en las historias clínicas en relación con los antecedentes familiares, personales y uso de medicamentos, ya que dichos datos son relevantes y podrían estar relacionados con el riesgo de disminución de la eTFG. Además, también hubo datos faltantes en el registro de los resultados de los exámenes de laboratorio que deben ser consignados en el sistema electrónico *Hygiaweb*. A pesar de ello, los datos obtenidos son los mismos utilizados para el monitoreo de las personas en uso de la PrEP en la práctica clínica y permitieron realizar un estudio de seguimiento de 48 semanas, tal como se encontró en otros estudios de evaluación de la función renal en personas en uso de la PrEP.

Entre las estrategias para ampliar el acceso al uso de la PrEP en los estados y municipios brasileños y alcanzar la meta de eliminación de la infección por VIH como problema de salud pública en Brasil, el Ministerio de Salud incluyó al enfermero como integrante del equipo de salud para prescribir la PrEP y realizar el seguimiento clínico de las personas usuarias de la PrEP, en el ámbito del SUS, conforme al Protocolo Clínico y Directrices Terapéuticas de la Profilaxis Previa a la Exposición al VIH (PCDT/PrEP), siempre que estén establecidos en programas de salud pública y en una rutina aprobada por la institución de salud^(9)^, con el objetivo de reducir el número de nuevas infecciones en consonancia con los ODS^(9)^.

Cabe resaltar que el reconocimiento de la competencia técnico-científica del enfermero expresa la relevancia de este profesional para el avance de las políticas de prevención y cuidado en el contexto de la infección por VIH, y configura una ampliación del alcance de la práctica clínica de Enfermería^(31)^. El enfermero debe incluir en cada consulta de enfermería con personas usuarias de la PrEP la evaluación de los marcadores de la función renal e implementar intervenciones de enfermería que involucren la literacidad en salud sobre la importancia del seguimiento clínico y de laboratorio, explicando la relevancia de la evaluación de la función renal, sobre todo entre las personas con mayor riesgo de alteración^(31-32)^, dado que las características sociodemográficas y clínicas de las personas son importantes para la implementación de estrategias de literacidad en salud personalizadas y adecuadas^(33)^.

Se destaca la importancia de la evaluación clínica de enfermería en el contexto de la prescripción de la PrEP y el acompañamiento integral de las personas en uso de la PrEP, incluyendo la solicitud de exámenes para el monitoreo adecuado de la función renal. El conocimiento clínico del enfermero para el monitoreo de la función renal es esencial para la identificación temprana de factores de riesgo y la conducción criteriosa de los procesos de inclusión, mantenimiento y eventual descontinuación del uso de la PrEP, asegurando la indicación de esta estrategia de prevención de manera adecuada y segura, considerando la singularidad clínica de cada persona^(34)^.

El monitoreo de la función renal de las personas en uso de la PrEP propuesto por este estudio tiene una contribución relevante para el avance del conocimiento científico, dado que aún hay pocos estudios que se centren en los efectos de la PrEP sobre los marcadores de la función renal a lo largo del tiempo, especialmente a nivel nacional, contribuyendo así a los consensos sobre el uso de esta importante estrategia de prevención. Este conocimiento refuerza las recomendaciones del protocolo brasileño y señala la importancia de intervenciones de salud y de enfermería enfocadas en el monitoreo y el autocuidado, particularmente entre las personas en uso de la PrEP que poseen factores de riesgo para disfunción renal.

## Conclusión

Las personas en uso de la PrEP que fueron acompañadas durante 48 semanas eran, en su mayoría, hombres, cis, homosexuales, de piel blanca, con alto nivel educativo y jóvenes, con una edad media de ± 34 años. La presencia de marcadores de daño renal predice una baja lesión renal en personas usuarias de la PrEP. Entre los marcadores, la proteinuria presentó un aumento hasta la semana 12 (2,6%), manteniendo una pequeña variación hasta la semana 48; todos los casos fueron eventos leves o grado 1 de proteinuria. La glucosuria fue aún más rara; sin embargo, un participante presentó evolución de grado 2 a grado 4.

En nuestro estudio no identificamos alteraciones en la media de creatinina sérica en la evaluación de las semanas de seguimiento; sin embargo, la eTFG calculada por Cockcroft-Gault presentó una disminución leve y progresiva en las semanas 12, 36 y 48, mientras que el CKD-EPI mostró reducción solo en las semanas 36 y 48. La eTFG calculada por CG, en comparación con el CKD-EPI, mantuvo una diferencia de alrededor de 20 mL/min durante todo el período del estudio, siendo la eTFG calculada por CG más alta que la calculada por CKD-EPI.

Además, los resultados de esta investigación ratifican la baja incidencia de eventos que caracterizan disfunción renal. Durante las 48 semanas de seguimiento del estudio, entre el 3% y el 4,4% de la población presentó una disminución de la eTFG igual o superior al 25%, además de eventos leves y aislados de proteinuria, glucosuria, uricosuria y cilindruria en personas usuarias de la PrEP. No hubo asociación significativa entre la disminución de la eTFG ≥25% y la presencia de alguno de los marcadores de daño renal estudiados.

## Data Availability

Los conjuntos de datos relacionados con este artículo estarán disponibles previa solicitud al autor correspondiente.
